# Leveraging tumor dynamics to discover mutations influencing progression and treatment response for precision oncology

**DOI:** 10.1186/s13073-026-01695-x

**Published:** 2026-06-17

**Authors:** Ella Petter, Jaclyn LoPiccolo, Stefan Groha, Richard Border, Bogdan Pasaniuc, Kenneth L. Kehl, Noah Zaitlen, Alexander Gusev

**Affiliations:** 1 Department of Computer Science, University of California, Los Angeles, Los Angeles, CA 90095 USA; 2 Department of Medical Oncology, Dana-Farber Cancer Institute & Harvard Medical School, Boston, MA 02215 USA; 3 Program in Medical and Population Genetics, Broad Institute, Cambridge, MA 02142 USA; 4 Division of Population Sciences, Dana-Farber Cancer Institute, Boston, MA 02215 USA; 5 The Broad Institute of MIT & Harvard, Cambridge, MA 02142 USA; 6 Department of Computational Biology, Carnegie Mellon University, Pittsburgh, PA 15213 USA; 7 Department of Genetics, Perelman School of Medicine, University of Pennsylvania, Philadelphia, PA 19104 USA; 8 Department of Biostatistics, Epidemiology and Informatics, Perelman School of Medicine, University of Pennsylvania, Philadelphia, PA 19104 USA; 9 Department of Computer and Information Science, School of Engineering and Applied Science, University of Pennsylvania, Philadelphia, PA 19104 USA; 10 Department of Computational Medicine, David Geffen School of Medicine, University of California Los Angeles, Los Angeles, CA 90095 USA; 11 Bioinformatics Interdepartmental Program, University of California Los Angeles, Los Angeles, CA 90095 USA; 12 Department of Human Genetics, David Geffen School of Medicine, University of California Los Angeles, Los Angeles, CA 90095 USA; 13 Department of Neurology, David Geffen School of Medicine, University of California Los Angeles, Los Angeles, CA 90095 USA; 14 Division of Genetics, Brigham and Women’s Hospital, Harvard Medical School, Boston, MA 02215 USA

**Keywords:** Precision oncology, Electronic health records, Somatic mutations, Tumor dynamics, Treatment response, Survival analysis, Longitudinal outcomes, Non-small cell lung cancer

## Abstract

**Background:**

Current research in precision oncology focuses on progression-free survival (PFS) and overall survival (OS) as the key outcomes. This stands in stark contrast to the primary outcomes guiding cancer treatment in the clinic- dynamic changes in tumor size in response to treatment. While PFS and OS are meaningful, highly interpretable and easily collected, we hypothesize that revealing associations between somatic mutations and tumor size dynamics can uncover genetic factors that can influence treatment response and generate hypotheses for future clinical investigation, while further improving our understanding of tumor biology.

**Methods:**

We propose and benchmark a new statistical framework – Andersen-Gill based Tumor Dynamics (AG-TD) to model recurrent outcomes and apply it to data from ~ 17 K radiology reports of patients with non-small cell lung cancer undergoing routine treatment at the Dana-Farber Cancer Institute. Our approach enables modeling recurrent outcomes while considering the time-dependent nature of biomarker-treatment interaction, patient mortality, and loss to follow-up.

**Results:**

We identify 27 somatic mutations that lead to differential response to common treatments including both protective and hazardous effects. Among these, deletions in *MYBL1* and *GATA3* were independently validated by replication in an external EGFR-TKI drug sensitivity screen, reaching Bonferroni correction significance. We also identify 13 somatic mutations associated with differential rates of progression and response during the entire treatment course, of which 9 are undetectable using conventional OS or PFS models. We further verify that the results are consistent in a homogeneous sub-cohort of advanced stage (3 and 4) patients only.

**Conclusions:**

Overall, we demonstrate the potential for using longitudinal outcomes in cancer genetic studies to complement traditional survival-based analyses, and to enable systematic identification of candidate gene–treatment interactions for future validation.

**Supplementary Information:**

The online version contains supplementary material available at https://doi.org/10.1186/s13073-026-01695-x.

## Background

Identifying and understanding the somatic features associated with disease progression and treatment response is a central objective of cancer research [1–3]. Such treatment biomarkers have facilitated the understanding of cancer biology, pathways [4–6] and clinical implementations [7–10], and have advanced the development of effective therapies [11–14]. Most biomarker discovery efforts have focused on single end-point outcomes such as overall survival (OS) or progression free survival (PFS) with relatively few attempts to use alternative or surrogate outcomes [15, 16]. Their interpretable nature, ease of collection, fundamental importance, and strong statistical foundation in survival analysis make them natural metrics of choice.

However, clinicians focus on more granular and longitudinal measurements that can capture additional components of the tumor trajectory [17] not visible with a single end-point or time-to-first-event analyses. These measurements include tumor size, symptoms, and the development of metastasis. For example, patients experiencing pseudo-progression [18] - a phenomenon where tumors initially appear to grow before subsequently showing a reduction in tumor size - are not accurately captured by a single time-to-first-event analysis. Moreover, as patients undergo multiple lines of therapy, single end-point analyses may start to capture the residual influence of prior therapy lines rather than the focal treatment of interest. While traditional end-points are undoubtedly important, particularly in clinical trials, we hypothesized that their limitations in biomarker studies may contribute to the reported lack of reproducibility [19–21] in the field and the difficulties in identifying new drug targets [22]. Indeed, in recent work [23], it was shown that contrary to earlier assumptions, biomarkers associated with OS were mostly housekeeping genes and not actionable for drug development, further demonstrating the need for new analytic approaches.

In this work we introduce a new paradigm for the identification of somatic variants associated with longitudinal tumor size variation on and off treatment, bringing research outcomes closer to those in the focus of clinical practice. By leveraging novel data from next generation sequencing (NGS) of tumor samples linked with electronic health records (EHR) and computational advances in the field of natural language processing (NLP) models [24], we propose to model the full trajectory of tumor size variation as a function of somatic variation. We focus on two longitudinal outcomes – tumor progression and response to treatment, capturing the worsening or improvement of the disease. We developed an analysis approach, Andersen-Gill based Tumor Dynamics (AG-TD) adapting Cox models [25] to tumor dynamics recurrent events while accounting for patient mortality, immortal time, and loss-to-follow-up [25, 26]. We further expand our approach to incorporate time-dependent drug information. This extension, xAG-TD, allows us to detect mutation-by-treatment interactions – somatic mutations associated with differential rates of response to specific drugs or treatments during patient treatment course.

We apply our approach to a cohort of non-small cell lung cancer patients (NSCLC) who were treated at the Dana-Farber Cancer Institute (DFCI). We detected 9 somatic mutations that have a significant association with tumor progression or response but are not detectable using standard Cox analysis for OS as an outcome. We further find 27 significant mutation-by-treatment interactions, for which a specific somatic gene mutation is associated with differential response to the same treatment. Each of these interaction associations is a natural candidate for further clinical investigation and experimentation as a target for precision oncology or companion diagnostics. More generally, our work presents a comprehensive statistical framework for the discovery of prognostic somatic mutations and mutation-by-treatment interactions in real-world EHR data [27, 28].

## Methods

### Dana-farber cancer institute data

This study used de-identified data from the Dana-Farber Cancer Institute (DFCI) electronic health record (EHR) database between 2011 and 2022. Our sample included 1,871 patients. Patients had an average age of 64.4 (SD 11.1), were 59.6% female, and spanned stage 1–4 at time of diagnosis (25.8%, 9.4%, 19%, 45.7% respectively). The average time of follow-up since diagnosis was 3.7 years (IQR 1.6–5.46 years). We captured 1,043 death records (55% of the patients in the cohort) and for patients who were alive at the time of data collection, the last date of contact was recorded. All patients in the study had NSCLC and had undergone next-generation sequencing as part of their routine treatment. The patients were sequenced with an OncoPanel [29], covering ~ 500 genes, with calling for single nucleotide variants (SNVs) as well as deletions and amplifications. We restricted the analysis to patients who were first diagnosed with cancer at DFCI and had at least 3 imaging reports in their EHR. The EHR includes information from an EPIC system regarding patients’ medications, as well as date of last contact and mortality records.

We set the anchor date as the date of diagnosis as recorded by DFCI for each patient. In each month, we aggregated all imaging records of the same patient and determined if they had a progression or response event in that month (see below). We removed any instances where the same month had both progressions and responses. Overall we captured 2,757 and 6,689 records of response and progression, respectively, out of ~ 17 K reports. We further removed patients who had a gap in their EHR of more than 3 years for which there were no imaging records. We excluded patients who were sequenced more than 10 years after their diagnosis as they were suspected to have a disease recurrence. Since tumor sequencing is an inclusion criterion in our study and is performed post-diagnosis, our data is susceptible to the so-called “immortal time bias”. In order to mitigate its impacts we performed left truncation until the sequencing date, introducing patients to the risk set post-sequencing only [30].

This patient cohort, including somatic mutation status, EHR data and longitudinal image-based tumor outcomes, was used for the main AG-TD and xAG-TD analyses.

### Extraction of longitudinal progression and response events

The recurrent progression and response events used in this analysis were obtained from free-text radiology reports in previously published and validated work [24]. In that work, NLP models were trained to extract clinically meaningful cancer outcomes, including progression and response, from routine imaging reports. The trained model achieved excellent agreement with human expert annotations (AUROC = 0.95 for progression and 0.97 for response). In the present study, these NLP-derived outputs were used to define recurrent progression and response events that serve as the model outcomes.

### Selection of treatments for analysis and extraction of medication data from EHR

We defined a list of common NSCLC drugs in the following categories: chemotherapy, immunotherapy and targeted drugs (Additional file 1: Table S1). We extract patient’s medication information from the EHR records directly. Each drug administration date is translated to be the month’s first date for unity. In case there are two different records of the same drug for the same patient within 3 months, we carry the drug information over the middle month as well. At last, for every patient we have monthly records capturing all the months where any drug was administered and which drug it was. We manually matched all the drugs to their regimen category and modified the data structure to also include the regimen category in each month. We filter out any medications that are not part of our monitoring list (Additional file 1: Table S1). We then match the medication data with the imaging encounters. In months where there is an outcome recorded but no drug was administered, we use the previous treatment, if it was less than a year before the imaging encounter date. This one-year maximal window aims to address irregular treatment documentation in the EHR rather than imply an assumption about persistent pharmacologic activity. Importantly, a sensitivity analysis using a substantially shorter window (100 days) yielded highly concordant results (correlation of log hazard ratios = 0.984, *p* < 2.2 × 10-16, see Additional file 1: Table S2). We only keep medication records that include a single type of drug from our list, or one of the combinations we defined. For example, we did not account for patients who receive EGFR inhibitors and chemotherapy [31] at the same time in either one of the categories, or in the background. This is because we have limited power to test all combinations unless they are very frequent. The combinations we allowed for are Cisplatin + Pemetrexed, Carboplatin + Pemetrexed and a combination of chemotherapy and immunotherapy drugs. All these combinations were considered as a separate treatments from their mono-treatment version. Other drugs not included in Additional file 1: Table S1 may be given at the same time and are ignored.

### Survival analysis with OS and PFS as outcomes

We used an OS and PFS Cox proportional hazards model [25] as the most simplified model, with diagnosis date as index time. We estimate the HR of each mutation on the overall patient survival or the time of first progression using a fixed set of baseline confounders, X. The baseline confounder set includes patient age, sex, tumor stage (as an indicator for advanced stages – 3 or 4, or early disease - stages 1 or 2), tumor mutational burden (TMB) and copy number burden (CNB) calculated by the total number of SNV and CNV mutations observed for each patient. Patients were followed until their death date or censored at their last contact date. The model is run separately for each somatic mutation, G, while considering SNVs, deletions and amplifications of the same gene separately:

$$\:h\left(t\right)={h}_{0}\left(t\right)\mathrm{e}\mathrm{x}\mathrm{p}({\beta\:}_{G}G+\sum\:{\beta\:}_{{X}_{i}}{X}_{i})$$

In this model, $h_0(t)$ is the baseline hazard at time t, and *h(t)* is the hazard function at time t. For each variable we obtain an HR value, which can indicate an increased hazard risk when its value is estimated over 1.

We use FDR multiple test correction [32] for each setting separately (outcome + gene + mutation type combination). To avoid biased estimates, we only run the analysis when we have more than 20 unique carriers of the gene tested.

### AG-TD model for association analysis with recurrent events

The standard AG-TD model is based on the Andersen-Gill model for recurrent events [33] as outcomes:

$$\rho\:\left(t\right)={\rho\:}_{0}\left(t\right)\mathrm{e}\mathrm{x}\mathrm{p}({\beta\:}_{G}G+\sum\:{\beta\:}_{{X}_{i}}{X}_{i}\left(t\right))$$

The outcome is either time-dependent progression or response to treatment as extracted from the radiology reports (see above), and their hazard is noted as $\:\rho\:$. When using response as an outcome we restrict events to those occurring while on active treatment (to differentiate between no response during treatment and stable no response event without treatment). This model also introduces a new covariate to the set of covariates *X*, capturing the time-dependent line number the patient is on at each time interval. In order to control for dependence of recurrent events of the same patient, we use the robust standard error estimation using the Huber estimate [34]. This approach provides valid statistical inference under within-patient correlation but does not alter the underlying mean effect estimate or fully account for potential residual event dependence. We use FDR multiple test correction for each setting separately (outcome + gene + mutation type combination).

### xAG-TD for detecting somatic mutation-by-treatment interactions

We extend our model to include the treatment information we curated from the EHR.

We iterate over all the treatments of interest, focusing on one treatment (*T*) at a time. We use the AG-TD model introduced above and include term *T* for the treatment effect and $\:T\times{G}$ for the interaction between the somatic mutation status and the treatment. The baseline confounders remain the same.

$$\begin{aligned}\rho\:\left(t\right)=&{\rho\:}_{0}\left(t\right)\mathrm{e}\mathrm{x}\mathrm{p}\left({\beta\:}_{G}G+{\beta\:}_{T}T\right. \\& +{{\upbeta\:}}_{\mathrm{I}}{T}\times{G}+\sum\:{\beta\:}_{{X}_{i}}{X}_{i}\left(t\right))\end{aligned}$$

The analysis was only run for treatment and mutation combinations where we had at least 10 distinct patients who are carriers and non-carriers in both the treatment and off-treatment groups, in addition to the previous set restriction of having at least 20 mutation carriers overall. In the analysis for treatment category interactions, when T is a treatment combination that combines immunotherapy (immunotherapy alone or combined with chemotherapy), we also include an interaction term between TMB and treatment *T*. After obtaining the p-values and coefficients for all the variables, covariates and interaction values, we perform multiple test correction using FDR on the interaction terms of each iteration separately. We report all the interactions with FDR < 0.05. The calculation and plotting of the MCF Nelson-Aalen estimate is done using the mcf function in the *reda: Recurrent Event Data Analysis* [35] package in R.

For the xAG-TD chemotherapy pairwise analysis we restrict patients to only be included in the model at times when they are given either one of the treatments of interest. We then remove some of the heterogeneity by changing the index time to be the first day of treatment instead of the day of diagnosis. We calculate the number of unique patients who are carriers and non carries, who have ever received the drug or never received the drug. We continue with the analysis only if in each of the four groups that are at least 10 unique patients. We perform multiple test correction using FDR on all the interaction term p-values. When reporting the significant results in Additional file 1: Table S3, we only consider results where both of the drugs in the pair had enough samples to complete the analysis, and report those with FDR < 0.05.

### Nelson-Aalen cumulative hazard function plots

Traditional Cox model results are often paired with visualizations based on Kaplan Meier curves [36]. Similarly, the AG-TD adaptation for such visualizations are Nelson-Aalen cumulative hazard function [37] plots, that can help interpret different interactions by comparing the hazard rates of different reference groups. These plots are independent from the AG-TD models and display the cumulative hazard function for different patient groups in our cohort – carriers and non-carriers, on and off the treatment of interest. These plots show the cumulative sum of the estimated probabilities for an event occurrence (also referred to as mean cumulative function) when the event is either progression or response:

$$\widehat{H}\left(t\right)=\sum\limits_{j=1}^{t}\frac{{d}_{j}}{{n}_{j}}$$

$\:\widehat{H}\left(t\right)$ represents the Nelson-Aalen cumulative hazard function, $\:{d}_{j}$ is the number of events occurring in the j-th time interval, and $\:{n}_{j}\:$is the risk set size at interval j, for all intervals preceding time t. The plots are based only on event times, not accounting for any confounders – making it suitable only for further examination of the signal but not for discovery, similar to Kaplan Meier plots in the Cox context.

### Replication of previous first-line treatment interactions

To allow for a proper comparison between results, we ran another analysis focusing on the effect of the interaction between somatic mutations and the patient’s first treatment category on OS. We first detect the first treatment category a patient received. We use the diagnosis date as index date, and the end date is the death date or date of last follow-up. We then run a classic Cox model to explain the survival time based on the patient’s age, sex, stage TMB, CNB as well as the first line treatment category, the carrier status of the mutation of interest and the interaction between the two. To match the analysis done by Liu et al.^28^ in which all mutation types (SNVs, deletions and amplifications) are considered the same, we combine all mutation types to get a single unified carrier status for each gene.

### Simulation framework for discovery under different genetic architectures

We conducted a simulation study to evaluate the effect of somatic carrier status, *G*, and non-growth mediated genetic factors, *N*, on the power of tumor growth, overall survival (OS), and progression-free survival (PFS). Our simulation cohort included 100 patients, each with 10 longitudinal tumor growth records. The genetic value of each patient, *G*, was determined by a binomial distribution with probability MAF-0.5. Longitudinal tumor growth records were generated based on their gene status. For patients with the gene (*G = 1*), tumor growth probability was set to $\:\mathrm{Pr}\left(growth\:\right|G=1)$. For those without the mutation (G = 0), it was set to $\:\mathrm{Pr}\left(growth\:\right|G=0)$. OS was then simulated by first calculating death probabilities over the 10 records of each patient. The probability of death at each record increased every time there was tumor growth event. To calculate death occurrence value at each time point for a given patient, we combined two components: the death probability due to tumor growth up to that point and a noise factor capturing the non tumor growth related effect of the gene status on survival, *N*. Specifically, the death event was computed as a weighted sum of the cumulative tumor growth-related death probability and a normally distributed random variable with a mean value, $\:\mu\:$ dependent on the gene status. The weights for these components were set to 0.5 each. PFS was determined by filtering records to identify the first occurrence of tumor growth or death. The simulation was repeated 100 times (simulating 100 genes) for each parameter combination, and the results were aggregated to calculate the power for detecting significant associations (*p* < 0.05) for OS, tumor progression, and PFS as the proportion of genes deemed significant.

In the first simulation set, we change $\:\mathrm{Pr}\left(growth\:\right|G=1)$ and $\:\mathrm{Pr}\left(growth\:\right|G=0)$, while keeping $\:\mu\:=0.2$ for both carriers and non-carriers, meaning the genetic influences on OS are fully mediated through tumor growth. We check the following combinations: (0.9, 0.2), (0.6, 0.2), (0.5, 0.3), (0.4, 0.3), (0.25, 0.2), (0.2, 0.2). In the second simulation we fix $\:\mathrm{Pr}\left(growth\:\right|G=1)=0.4$ and $\:\mathrm{Pr}\left(growth\:\right|G=0)=0.3$, and consider a grid of combinations for $\:\mu\:|G=1$ and $\:\mu\:|G=0$: (0.2,0.2), (0.3,0.2), (0.4,0.2), (0.5,0.2), (0.6,0.2), (0.7,0.2), slowly increasing the direct, non growth mediated effects of genetics on OS. We further look at the combination of for $\:\mu\:|G=1=0.4$ and $\:\mu\:|G=0=0.2$, and compare the significant (FDR < 0.05) p-values obtained for the 100 genes under each outcome model.

In the last set of simulations, we investigated a scenario where the AG-TD model correctly reports a true negative while the OS model falsely reports positives. In this scenario, the somatic mutation is uncorrelated with progression or death but is linked to delayed diagnosis. We used 5 K patients. We simulated 36 monthly intervals for each patient, with a constant rate of progression (5% probability of a progression event). The death rate had a base of 1%, increasing by 5% for each cumulative progression event experienced by the patient. Somatic mutation status was randomly assigned with a MAF of 50%.

For each patient, we selected a monthly interval for diagnosis using a normal distribution with different means for patients with and without the mutation (mean = 5 and 2 months, respectively, with a standard deviation of 2 months). Diagnosis intervals were constrained to be within the valid range of 1 to 36 months. Data was further censored at death and left truncated to the interval of diagnosis.

### *EGFR* epistatic effect analysis

For the post-hoc examination of the xAG-TD associations discovered of non-*EGFR* mutations with the EGFR treatment we first created a sub-cohort of patients who had any type of mutation (SNV, deletion or amplification) in the *EGFR* gene (*n* = 200). We then restricted the data to only include time intervals when EGFR treatments were administered to the patients. We run the standard AG-TD model again on this sub cohort without adding any interaction.

### Somatic mutation - drug association replication in DepMap and CTRPv2

We validated our associations using two experimental datasets. First, we used the DepMap dataset [38, 39], combining drug sensitivity data from the PRISM Repurposing Public 24Q2 release with copy number data from the 24Q4 release [40]. We restricted the dataset to non-small cell lung cancer cell lines. For each gene of interest, we selected cell lines with an absolute copy number ≤ 1 for deletions, and ≥ 2 for amplifications. We then performed a linear regression for each gene-drug pair, modeling the log fold change (LFC) in cell line abundance after drug treatment, as observed in PRISM as a function of the continuous copy number value for each cell line, where more negative LFC values indicate greater drug sensitivity. We report the slope and p-value of each combination. To estimate a pan-drug effect for each gene, we performed a fixed-effect meta-analysis across drugs using the rma function from the R metafor [41] package, incorporating the individual slopes and their standard errors.

Second, we conducted an analogous analysis using CTRPv2 [42–44] drug response data, in which drug sensitivity was quantified by area-under-the-curve (AUC) values derived from dose–response assays. For each gene-drug pair we performed a linear regression of the AUC as a function of the continuous copy number value in each cell line, and report the slope and p-value. The pan-drug effect was evaluated using the fixed-effect meta analysis approach by using the rma function from the metafor R package, incorporating the individual slopes and their standard errors.

### Simulation of response – OS correlation with logHR estimation errors

To assess the impact of estimation error on the observed correlation between response and OS logHRs, we performed a simulation based on the logHR standard errors observed in real data. We began with the observed OS logHR estimates across all somatic mutations and generated a second vector of latent response logHRs with prespecified Pearson correlations to the OS logHRs (r ∈ {-1, -0.75, -0.5, − 0.25, -0.1}). Latent response effects were constructed as a linear combination of the OS logHRs and an independent noise term, scaled to achieve the desired correlation. Estimation uncertainty was then incorporated by adding independent Gaussian noise to both OS and latent response logHRs, with standard deviation equal to the robust standard error estimated for each original logHR in the real data. For each target correlation value r, this procedure was repeated 500 times, and the Pearson correlation between the noisy OS and response logHRs was computed. The distribution of observed correlations across replicates was used to quantify attenuation of correlation magnitude due to uncertainty in logHR estimation, and the proportion of replicates with statistically significant correlations (*p* < 0.05) was used to assess power under the observed noise levels.

## Results

### Overview of data

**Fig. 1:**
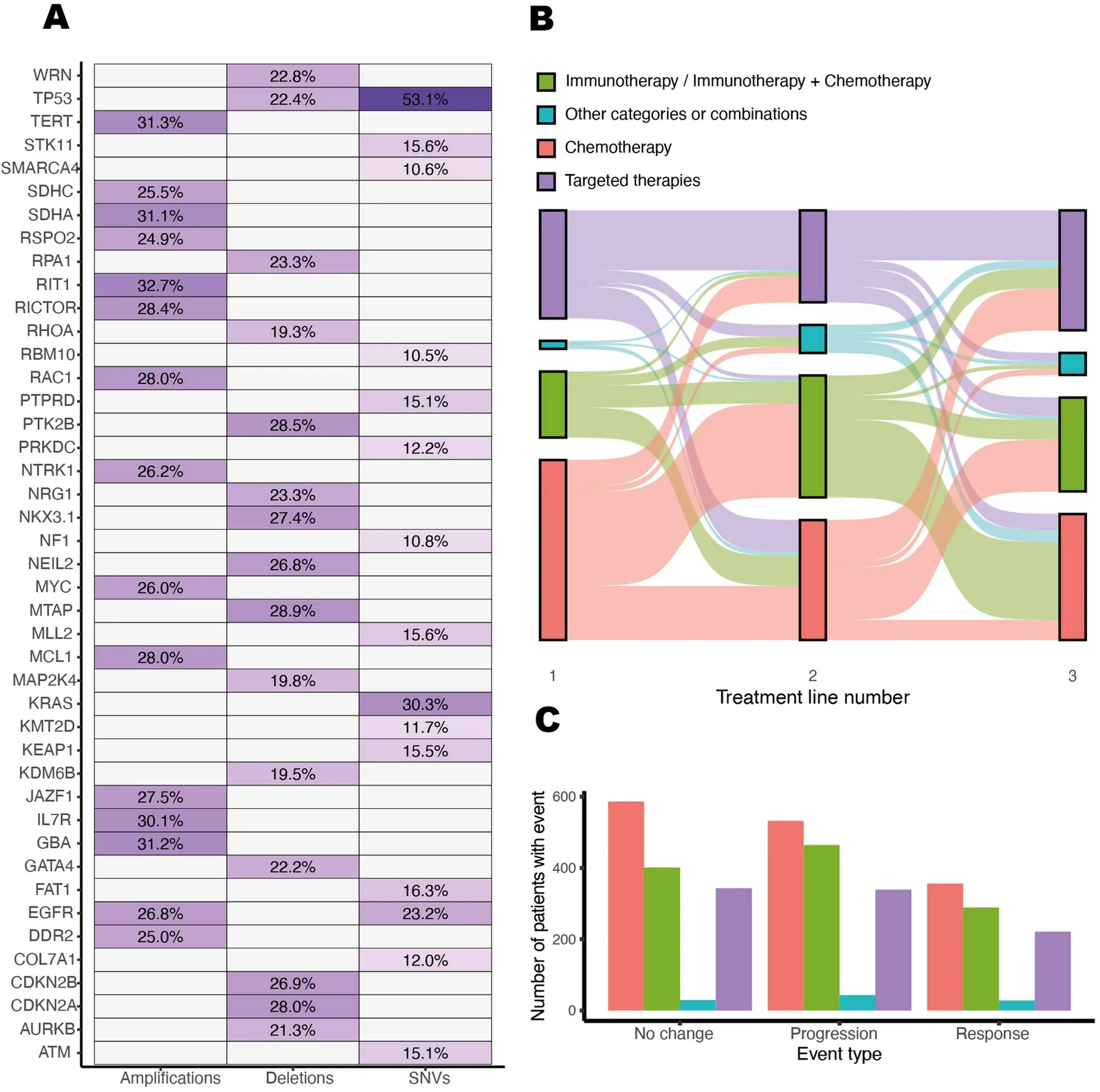
Data overview. **A** The proportion of carriers in the cohort for the top 15 most prevalent SNVs, amplifications and deletions. **B** Sankey plot demonstrating the changes between treatment types for 181 patients for whom we have image reports during their 3 first lines of treatment. **C** Number of patients with each treatment category and event type across the first three treatment lines

We studied 1,871 NSCLC patients treated at the Dana-Farber Cancer Institute (DFCI), who underwent clinical tumor sequencing [45]. Each patient had a paired EHR record, capturing imaging reports from which we extracted longitudinal cancer outcomes. Using a deep natural language processing algorithm (see Methods), we map every recorded image report to an event type – tumor progression, response or no change. Overall, across all patients, we captured 2,757 and 6,689 records of response and progression, respectively, as well as 1,043 death records. We observe that gene amplifications were more common than deletions and SNVs, but this may reflect amplifications across entire arms covering multiple genes. Figure 1a displays the proportion of patients carrying the 15 most prevalent mutations of each type.

The patients in our cohort were administered a wide range of therapies. We focused on common treatments for NSCLC including chemotherapy regimens, immunotherapy, and targeted therapies such as ALK and EGFR inhibitors (Additional file 1: Table S1). Out of the patients in our cohort that were administered at least one drug we account for, 60% have a change in their treatment regimen over the course of their documented treatments. Looking specifically at a sub cohort of 181 patients who have at least 3 documented treatment lines at times covered by their imaging reports (Fig. 1b), we see a strong flow of patients treated with chemotherapy and switching to immunotherapy or targeted therapies for their advanced lines. It is of note that in conventional biomarker studies such dynamic treatment changes are difficult to account for or require restricting to only first line treatments at the cost of a substantial number of observations.

Among patients receiving one of the first three treatment lines, progression and no-change events were observed at similar frequencies, while response events were less common (Fig. 1c). Each patient contributed at most one record per treatment category and event type. We observe the distribution of different treatments is similar across all event types.

### Overview of the AG-TD recurrent event model

**Fig. 2:**
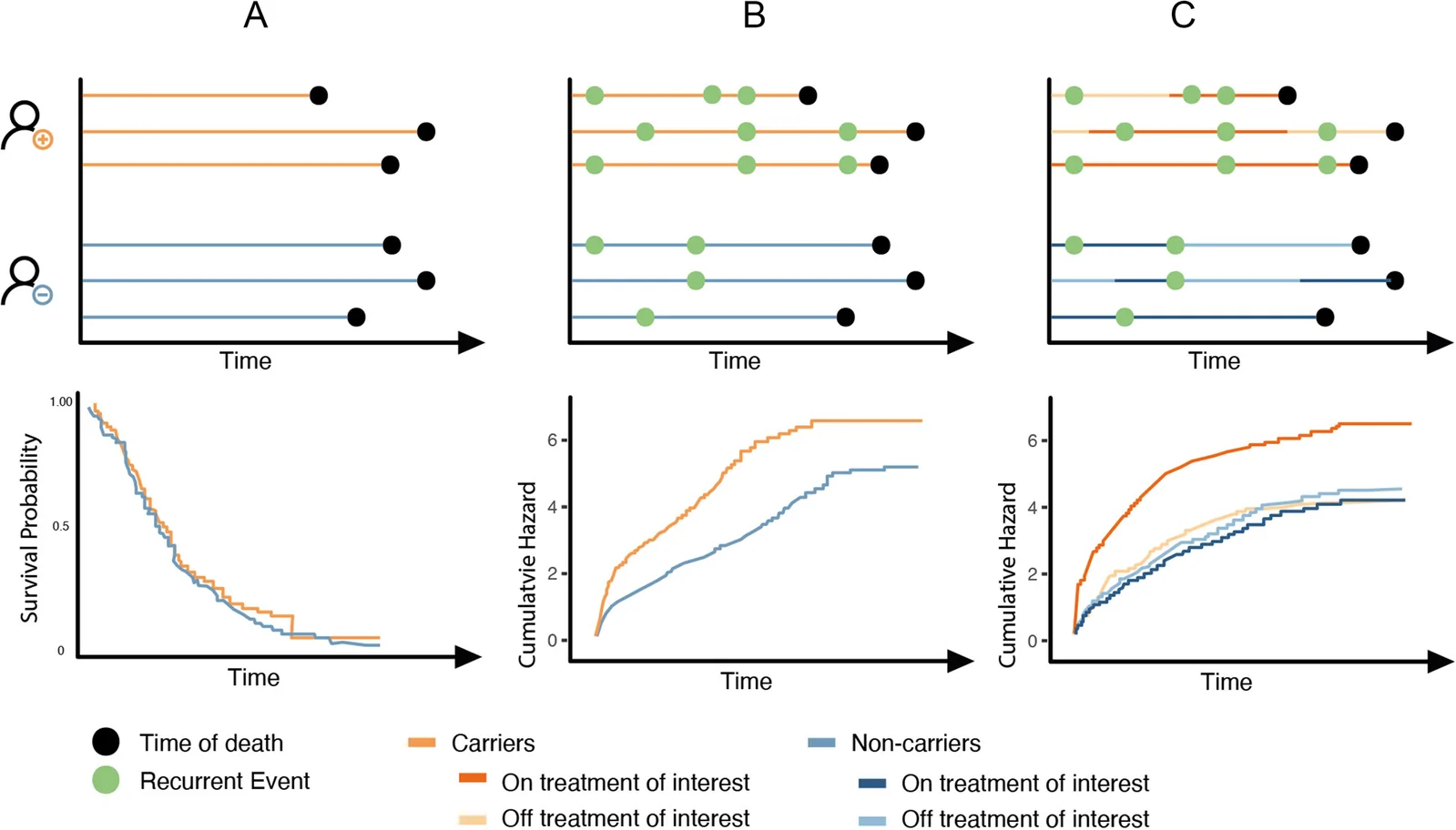
Overview of time dependent survival analysis in cancer. **A** traditional association studies (e.g. Cox) link somatic carrier status with a single time point - usually PFS or OS, and visualize results using Kaplan-Meier plots (bottom). **B** with AG-TD, we utilize EHR longitudinal outcomes of progression and response, harnessing the full power of the data and avoiding noise from other factors affecting survival, and visualize results using the Nelson-Aalen cumulative hazard functions. **C** the xAG-TD model allows for further stratification by treatment type, revealing interactions that are undetectable otherwise

We present an overview of our model for identifying treatment-interacting effects of somatic mutations on recurrent events of disease progression or response (Fig. 2). To facilitate understanding, we first introduce the conventional survival model and then extend to recurrent events and interactions.

The Cox proportional hazards model is widely used for studying the impact of somatic mutations on single-event outcomes [25] by relating carrier status to survival through a hazard function over time (* t*):

1 $$h\left(t\right)={h}_{0}\left(t\right)\mathrm{exp}\left({\beta\:}_{G}G+\sum\:{\beta\:}_{{X}_{i}}{X}_{i}\right)$$

where, $\:{h}_{0}\left(t\right)$ represents the baseline hazard at time * t*, * G* is a binary variable indicating the presence of a somatic mutation of interest, and $\:{X}_{i}$ represents other covariates, such as age or tumor stage. In this context, the hazard refers to the probability of the event (in this case, death) to happen at a time * t*, assuming the patient has survived up to * t*. Individual effect sizes ($\:\beta\:)$ for each variable, also known as log Hazard Ratios (HR), are then estimated using Cox regression. For example, if $\:{\mathrm{e}\mathrm{x}\mathrm{p}(\beta\:}_{G})=2$, the interpretation is that carriers of the gene of interest have twice the hazard of non-carriers, whereas $\:{\mathrm{e}\mathrm{x}\mathrm{p}(\beta\:}_{G})=1$ corresponds to equal hazards.

The conventional Cox model treats all events as terminal, whereas we are interested in identifying somatic mutations that affect the rate of recurrent tumor events in a specific treatment context. For this we propose a new framework, AG-TD (Fig. 2b), based on the Andersen-Gill model for recurrent survival outcomes [33, 46–48]. AG-TD allows one to use multiple recurrent outcome observations for each individual, and to incorporate time-dependent variables. For intuition, a biomarker that influences a recurrent outcome can be thought of as increasing the rate at which the event occurs (e.g. a somatic mutation that increases the rate at which the tumor grows) or reducing the mean interval time between events. In contrast to model (1), the AG-TD hazard function of recurrent events is given by:

2 $$\rho\:\left(t\right)={\rho\:}_{0}\left(t\right)\mathrm{e}\mathrm{x}\mathrm{p}({\beta\:}_{G}G+\sum\:{\beta\:}_{{X}_{i}}{X}_{i}\left(t\right))$$

In Eq. (2), the variable $\:{X}_{i}\left(t\right)$ represents clinical covariates (some time-varying) such as age, sex, stage or line of therapy. $\:\rho\:\left(t\right)$ and $\:{\rho\:}_{0}\left(t\right)$ are the hazard and baseline hazard for the recurrent event of interest at time t. The baseline hazard is common for all recurrences, and the recurrent event times are assumed to be independent within an individual. The main conceptual adaptation in this model is that every patient is represented by multiple time intervals corresponding to different values of the event’s occurrence. At each time point, the cohort will include patients who are in the risk set (e.g. not censored or left truncated at the time), and patients remain in the risk set after experiencing the recurrent event until death or loss to follow-up (both treated as censoring events). To account for dependencies between events from the same patients, a robust standard error is calculated using the Huber estimator [34].

We are additionally interested in identifying biomarkers that may be predictive of specific treatment benefit, i.e. exhibit treatment interactions. Such biomarkers exhibit significantly different hazard ratios in the context of different treatments. For instance, tumors with a mutation in the EGFR gene tend to grow faster during chemotherapy but slower during EGFR inhibitor treatments. Treatment interactions can thus capture optimal treatment regimens, rather than general prognostic influences that do not differ by treatment. To facilitate such interaction discovery, we propose the xAG-TD model (Fig. 2c) as an expansion of the AG-TD model. The xAG-TD model specifically accounts for the treatment-interacting effect of somatic mutations on recurrent events related to disease progression or response:

3 $$\begin{aligned}\rho\:\left(t\right)=&{\rho\:}_{0}\left(t\right)\mathrm{e}\mathrm{x}\mathrm{p}\left({\beta\:}_{G}G+{\beta\:}_{T}T\right. \\& +{{\upbeta\:}}_{\mathrm{I}}T\times{G}+\sum\:{\beta\:}_{{X}_{i}}{X}_{i}\left(t\right))\end{aligned}$$

xAG-TD includes a new binary time-dependent variable, * T*, equal to 1 at time intervals where the patient was given treatment * T*. This model further includes the interaction between the treatment status and the presence of a somatic mutation. By estimating the effect size for the interaction $\:{{\upbeta\:}}_{\mathrm{I}}$, we can learn its effect on the patient hazard. For example, if $\:{{\upbeta\:}}_{\mathrm{I}}$ is significant and has a value larger than 1, it means that patients on the treatment of interest * T* have a higher event rate if they are also carriers of * G*, compared to non-carriers of mutation * G*, or alternatively, that carriers of gene * G* on treatment * T* have a higher event rate than carriers on alternative treatments. We note that traditional Cox model results are often paired with Kaplan-Meier curve visualizations, while the AG-TD adaptation uses Nelson-Aalen [37] cumulative hazard function plots to help interpret interactions by comparing hazard rates of different reference groups (Fig. 2b, c, bottom panels, see Methods).

Throughout this manuscript we present results obtained by the AG-TD and xAG-TD models, using different recurrent outcomes – progression and response, and different mutation types – deletions, amplifications and SNVs. In the xAG-TD analysis, each treatment *T* is run in a separate iteration of the model. We use day of diagnosis as day 0 for all patients, and perform risk set adjustment by left truncating patients until their sequencing time in order to account for immortal time bias [30]. The covariates used are patient age, sex, tumor stage, tumor mutational burden (TMB) and copy number burden (CNB) calculated by the total number of SNV and CNV mutations observed for each patient. Patients were followed until their death date and censored at their last contact date or death date.

### Somatic mutation interactions with specific chemotherapy drugs

We first sought to identify somatic mutations whose interaction with specific chemotherapy drugs have a significant effect on the rate of progression or response events. We curated a list of 8 common chemotherapy regimens administered for NSCLC patients at DFCI (Additional file 1: Table S1) and restricted our cohort to 3534 recurrent events of 707 distinct patients during their chemotherapy treatment. Five out of the eight chemotherapy treatment types are single agent chemotherapies, and three are combinations (Carboplatin + Pemetrexed, Cisplatin + Pemetrexed, Carbo + Paclitaxel). We include patients who are on any of these regimens exclusively, not in combination of immunotherapy or other targeted therapies. We ran the cross-chemotherapy xAG-TD model multiple times, each run independently focusing on a specific gene and mutation type of interest, as well as a specific treatment of interest. In each regression we measured the effect size and significance of the interaction between the time-varying treatment and mutation, $\:{{\upbeta\:}}_{\mathrm{I}}$, with the broader goal of finding mutations associated with differential event rates in treatment and non-treatment groups. We also report the value of $\:{{\upbeta\:}}_{\mathrm{G}}$ from the interaction model and from the main, treatment agnostic model. The $\:{{\upbeta\:}}_{\mathrm{G}}$ value from the interaction model should be interpreted as the additional hazard in gene G carriers, while $\:{{\upbeta\:}}_{\mathrm{I}}$ is the additional risk added for carriers who are also on the treatment T. The $\:{{\upbeta\:}}_{\mathrm{G}}$ value from the standard AG-TD model does not differentiate between treated and non-treated individuals. Notably, in many cases, the main gene effect $\:{{\upbeta\:}}_{\mathrm{G}}$ is not statistically significant, while the corresponding gene-treatment interaction term $\:{{\upbeta\:}}_{\mathrm{I}}$ is.

**Fig. 3:**
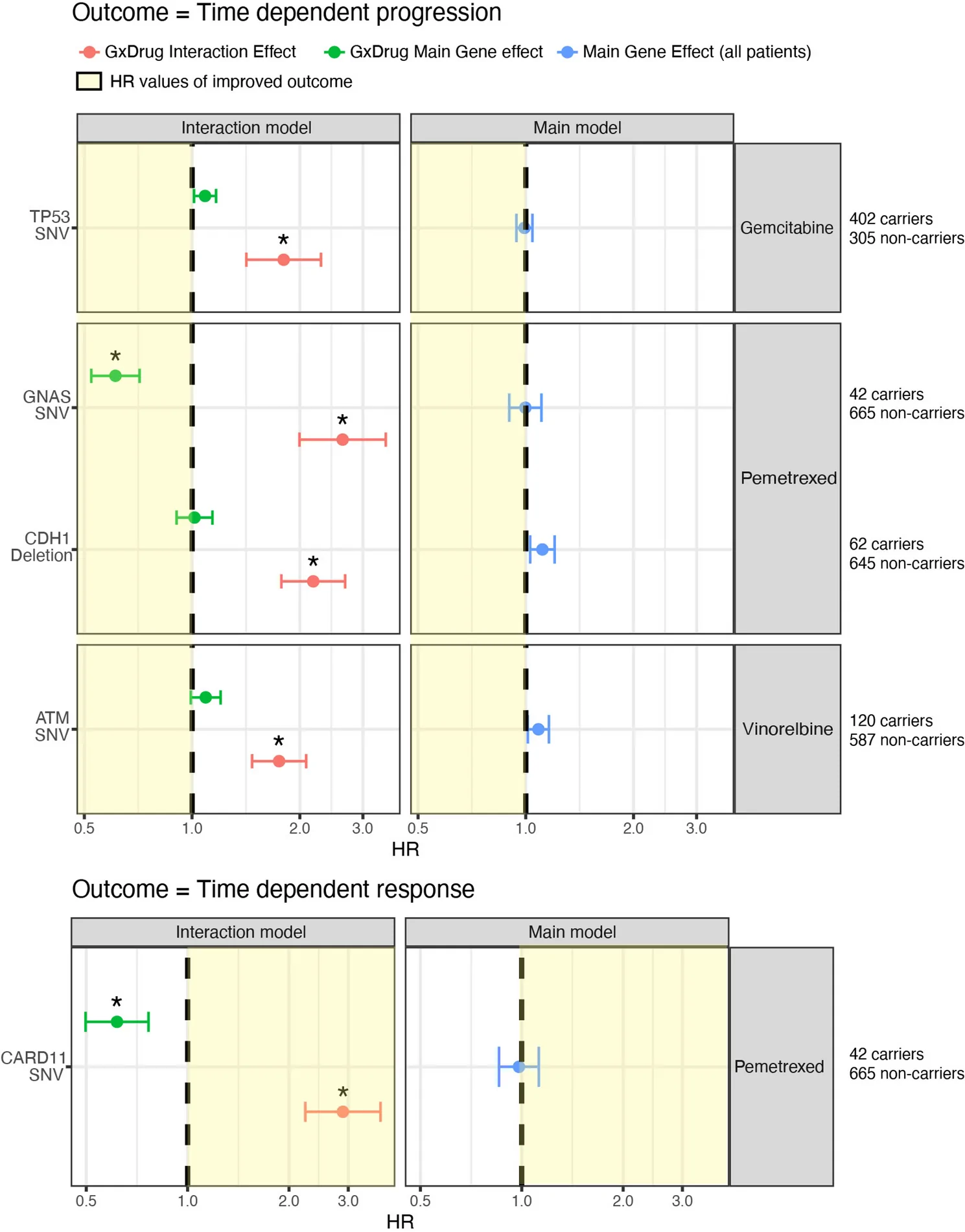
Effects of somatic mutation interactions with chemotherapy regimens on patient progression and response. HR (on log_10_ scale) and SE obtained for interactions between somatic mutations and specific chemotherapy regimens. The green and blue points mark the HR obtained for the same mutation type as a main effect in the interaction model, and in a non-interaction model, respectively. When they are nominally significant, they are further marked with (*). The red points represent the HR for the interactions, and they are all significant (FDR < 0.05). The shaded yellow area corresponds to the better outcome (i.e. less progression or more response)

**Fig. 4:**
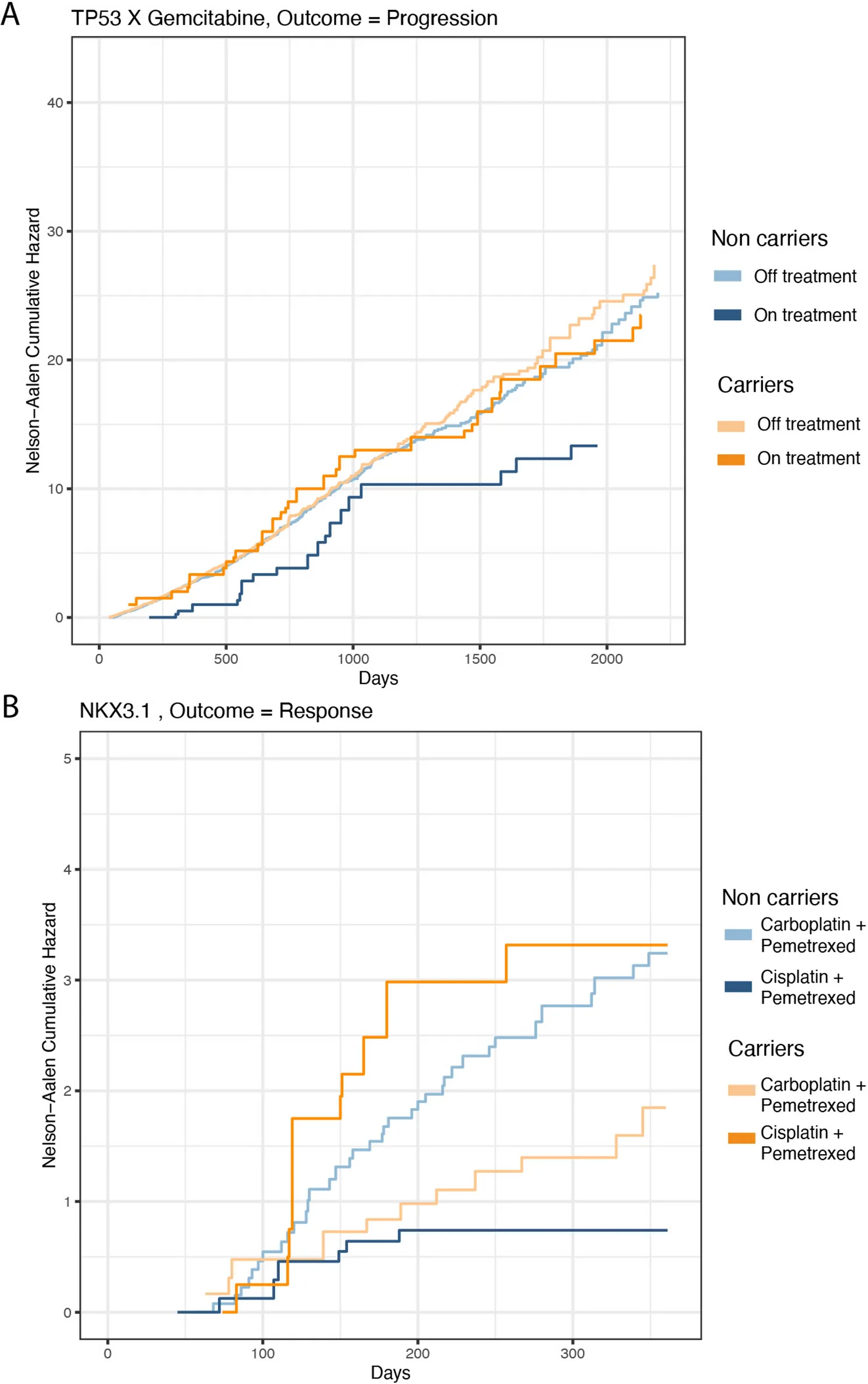
Cumulative hazard function of recurrent events for a sub-cohorts of carriers and non-carriers, on and off treatments of interest. **A** The plots represent the cumulative sum of recurrent progression probabilities over time. Patients are split into 4 groups – non-carriers (blue) and carriers (orange), while they are on treatment (darker colors) or off the treatment of interest (lighter colors). The X axis shows the days since diagnosis, and the Y axis shows the cumulative hazard function estimate as obtained by the Nelson Aalen estimate. While non-carriers of *TP53* SNV have lower rates of progression under Gemcitabine treatment, this effect is not preserved to carriers who have the same progression rates while on and off Gemcitabine regimen. **B** In this sub-cohort patients are either on Cisplatin + Pemetrexed (darker colors) or Carboplatin + Pemetrexed (lighter colors). In *NKX3.1* non-carriers, the Carboplatin yields improved results (higher cumulative response) while for carriers, Cisplatin results in improved outcome

We detected 5 genes in which somatic mutations have an FDR < 0.05 significant interaction with the chemotherapy type (Fig. 3). Out of these associations, 4 were SNV mutations and 1 was a CNV deletion. One of the interactions detected is between *TP53* SNVs, the most common mutation type in our cohort, and Gemcitabine. The estimated $\:{{\upbeta\:}}_{\mathrm{I}}$ HR was 1.8 (p-value 1.4 × 10^− 2^, FDR corrected p-value 2.7 × 10^− 2^). This result can be interpreted as the hazard for *TP53* mutation carriers receiving Gemcitabine includes an additional multiplicative factor of 1.8 beyond the main effects of *TP53* mutation status ($\:{{\upbeta\:}}_{\mathrm{G}}$) and Gemcitabine treatment ($\:{{\upbeta\:}}_{\mathrm{T}}$), corresponding to the estimated interaction term. At 1-year since diagnosis, the observed mean cumulative hazard of *TP53* SNV carriers on Gemcitabine was 3.3 (SE 0.46) compared with a mean cumulative hazard of 1 (SE 0.42) for non-carriers on Gemcitabine (see Fig. 4), whereas carriers off treatment and non-carriers off treatment had a 1-year mean cumulative hazards of 2.67 (SE 0.18) and 2.67 (SE 0.22) respectively. While the cumulative hazard calculation is a different statistical approach to estimate hazards, these results are consistent with xAG-TD interaction term (Fig. 3). Notably, the main effect of being a *TP53* carrier did not have a significant HR on its own in the AG-TD model, and only adding the interaction revealed the underlying association between *TP53* and Gemcitabine. This result directly generates a clinical hypothesis which could be validated in a clinical trial where *TP53* wild-type tumors are assigned Gemcitabine treatment and *TP53* mutant tumors are assigned an alternative chemotherapy treatment. As a sensitivity analysis we ran the analysis on only the sub-cohort of advanced stage patients (stages 3–4). As this reduces sample size, we were only able to test 4 out of 5 associations, but all four were nominally significant (p-value < 0.05) and in the same direction of effect in the advance stage cohort (Additional file 1: Table S4).

In routine clinical practice, several chemotherapy regimens are considered clinically interchangeable, and there are no established biomarkers to guide the choice between them. As a result, treatment selection among these equipoise drug pairs is often driven by physician preference, logistical or toxicity considerations rather than predicted efficacy. This provides an opportunity to evaluate whether somatic mutations are associated with differential benefit between otherwise interchangeable regimens. We focused on finding somatic interactions between equipoise treatment pairs - treatments that are considered interchangeable and administered to patients with similar clinical features and as parallel treatment lines. We selected two pairs of chemotherapy regimens that are considered to be equipoise for NSCLC – the first is Vinorelbine vs. Gemcitabine and the second is Cisplatin + Pemetrexed combination treatment vs. Carboplatin + Pemetrexed combination treatment [49]. In this analysis we only included time intervals when patients received one of the drugs in the pair of interest (and no other drug from the drug list we account for as detailed in Additional file 1: Table S1), and the index time is set to the initiation of the chemotherapy treatment. Seven significant (FDR < 0.05) associations were detected, all for response as an outcome and for gene deletions whose carriers had higher response rates under the chemotherapy combination of Cisplatin + Pemetrexed compared to Carboplatin + Pemetrexed (Additional file 1: Table S3). Importantly, these associations reflect differential outcomes between the two regimens ionalional on genomic status, rather than absolute sensitivity to either platinum agent alone. Further exploration of these genes reveals they consist of two gene groups in which deletions are highly correlated. The first group is *SMAD2*, *SMAD4* and *SETBP1*, all located on the long arm (q) of chromosome 18 (pairwise Pearson correlation > 0.9). The strongest association amongst the genes in this group was the interaction of Carboplatin + Pemetrexed and *SMAD4* (HR 0.379, nominal p-value 3.4 × 10^− 3^). We find that the marginal logHR of non-carriers is larger by 0.77 when treated with the Carboplatin + Pemetrexed, however, in carriers it is 0.2 less than when on Cisplatin + Pemetrexed. This suggests that the optimal choice for deciding between these two treatments is carrier status dependent. These results may be related to previous findings of SMAD gene family connections to the TGF-β pathway and chemosensitivity to Cisplatin [50–52].

The second gene group is *GATA4*, *NEIL2*, *NKX3.1* and *PTK2B*, all located on the short arm (p) of chromosome 8 (pairwise Pearson correlation > 0.88). The strongest association in this group was observed for the interaction of *NKX3.1* with Carboplatin + Pemetrexed treatment (HR 0.154, nominal p-value 2.6 × 10^− 4^). Similar to the previous group, among non-carriers of *NKX3.1*, Carboplatin + Pemetrexed shows greater effectiveness, with a marginal logHR that is 1.63 higher for response compared with Cisplatin + Pemetrexed treatment in non-carriers. In carriers of *NKX3.1*, however, the result reverses: their marginal logHR is 0.24 lower with Carboplatin + Pemetrexed compared to Cisplatin + Pemetrexed. This signal may be related to initial results demonstrating the effect of the overexpression of the Pyk2 protein encoded by *PTK2B* inducing resistance to Cisplatin in liver cancer [53], however importantly, this study does not evaluate carboplatin as a head-to-head treatment comparisons, and therefore does not directly inform the direction of comparative effects observed here.

To further evaluate whether these correlated gene signals represent independent effects or a shared CNV-driven event, we performed a conditional interaction analysis within each chromosome arm. For each chromosome arm, we included the main effects of all genes in the arm to account for co-occurrence and retained the treatment interaction term only for the top gene based on the strongest interaction term from initial results (*SMAD4* in the 18q arm, *NKX3.1* in the 8p arm). In both cases, the interaction between treatment and the top gene remained significant (*SMAD4* interaction p-value = 3.8 × 10⁻³; *NKX3.1* interaction p-value = 4.9 × 10⁻⁴), while all gene main effects were non-significant. These results indicate that the treatment interaction cannot be explained by the co-occurrence of other CNVs along the chromosome arm, whereas the most significant interaction explains all of the marginal CNV effects. This is consistent with a single underlying event driving the treatment interactions, though we cannot definitively identify the causal element given the high correlation of CNV carrier status within each arm.

Given the restricted sample size of the equipoise xAG-TD analysis, we aimed to explore whether the hazard ratios (HR) obtained from that analysis correlate with a broader cross-chemotherapy xAG-TD analysis. Our motivation was to determine if the more robust cross-chemotherapy xAG-TD analysis, which includes multiple treatments and a different treatment initiation, could still provide generalizable associations to the more specific equipoise pairwise analysis. We compared the logHR values of 43 nominally significant (p-value < 0.05) cross-chemo xAG-TD interactions that also had corresponding equipoise results. The Pearson correlation between the logHR values of the cross-chemo xAG-TD pairs and the equipoise xAG-TD pairs was 0.948. Additionally, 62.7% of these interactions were also nominally significant in the equipoise pairs xAG-TD analysis (see Additional file 2: Fig. S1). These findings suggest that results from the more general cross-chemotherapy xAG-TD analysis can serve as reliable predictors for the logHR values in matching equipoise xAG-TD analyses.

### Somatic mutation interactions with broad treatment regimens

We next sought to identify mutations whose interactions with broad treatment categories (e.g. chemotherapy, immunotherapy, etc.) were associated with recurrent outcomes. While these treatments are often administered in a structured order based on stage and prior therapy, biomarker interactions may motivate novel clinical trial designs or point to novel biological mechanisms.

As a positive control, we evaluated the effect of the interaction between tumor mutational burden (TMB) and receiving immunotherapy treatment on cancer progression. We found the interaction between high TMB (defined by having TMB above the cohort mean of 12, see Additional file 2: Fig. S2) and being treated with immunotherapy was significant for reduced progression rates (HR 0.7, *p*-value 1.6 × 10^− 3^). This result is in line with the use of high TMB as a biomarker for better outcomes with immunotherapy treatement [54, 55]. It is of note that this result did not replicate with the PFS/OS version of this analysis as the interaction between high TMB and ever receiving immunotherapy (binary, non time dependent) was non-significant. We include an interaction term with TMB for all subsequent interaction analysis with immunotherapy due to the possible confounding between gene mutations and elevated TMB levels.

Another positive control we evaluated is the interaction between *EGFR* mutation carriers and EGFR treatment. Using xAG-TD, we find the interaction between EGFR treatment and *EGFR* SNV has a significant reduction effect on progression (HR of 0.45, p-value 1.4 × 10^− 12^, FDR *p*-value 4.3 × 10^− 11^). It is important to note that this analysis includes 862 non-carriers and 279 carriers, out of which 2% and 71% were ever administered EGFR treatment, so it is probable much of the signal comes from EGFR carriers during different lines of treatment other than EGFR inhibitors. For comparison, we conducted a matching analysis using OS and PFS as outcomes to estimate the effect of *EGFR* carrier status interaction with ever receiving EGFR treatment. The OS association was significant (*p*-value 0.02, HR 0.52), and the PFS association was even stronger (*p*-value 6.6 × 10^− 4^, HR 0.32), however the xAG-TD signal obtained was stronger than both. These positive controls highlight how xAG-TD can substantially increase the statistical power to detect causal treatment interactions.

**Fig. 5:**
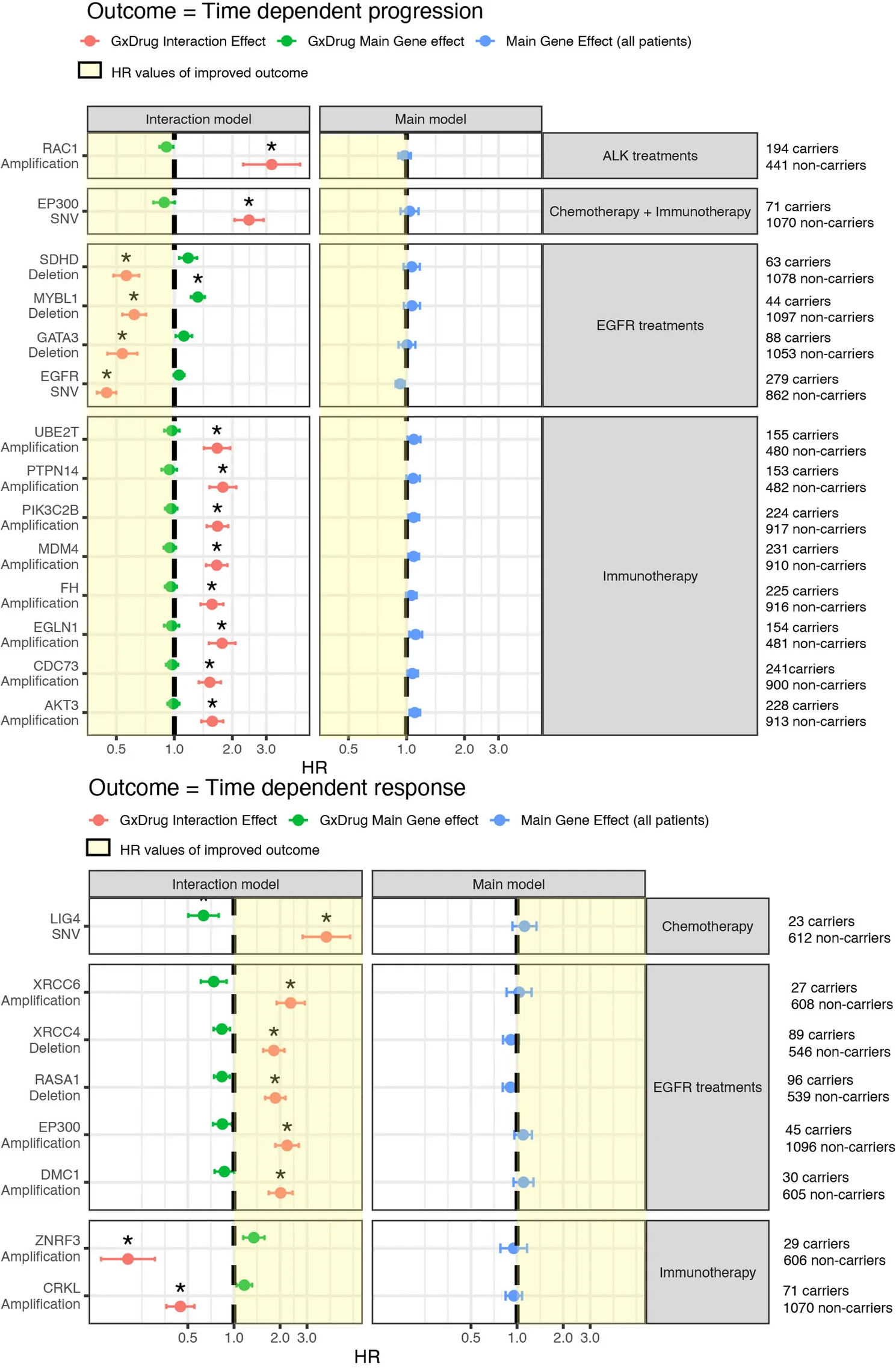
Effects of somatic mutation interactions with treatment type on progression and treatment response. HR (on log_10_ scale) and SE obtained for interactions between somatic mutations and specific treatment regimens. The red points represent the HR for the interactions, and they are all significant (FDR < 0.05). The green and blue points mark the HR obtained for the same mutation type as a main effect in the interaction model, and in a non-interaction model, respectively. The shaded yellow area corresponds to the better outcome (i.e. less progression or more response).

For response and progression separately, we ran 1291 iterations of the analysis, each for a different combination of mutation of interest and treatment category: chemotherapy, immunotherapy, combined chemotherapy/immunotherapy regimen, ALK inhibitors, EGFR inhibitors and other targeted therapies. We found 14 somatic mutations whose interactions with a treatment category were significantly associated (FDR < 0.05) with the hazard for disease progression events and 8 for response events (Fig. 5). Some of these CNV mutations were found to have a highly correlated signal and were in physical proximity, suggestive of broader (e.g. arm-level) events. In such cases, our method does not distinguish between tagging and causal signal. An example of such correlated arm association involves an amplification of the q arm of chromosome 1 including genes *PIK3C2B*, *AKT3*, *MDM4*, *FH*, *UBE2T*, *EGLN1*, *CDC73*, and *PTPN14* associated with increased progression rates with immunotherapy (Fig. 5). We performed a conditional analysis for these mutations, testing if the most significant interaction association remains significant after adjusting for the main effect of other correlated genes. In this case, *PIK3C2B* was the most significant interaction gene initially, and after including a variable for each other gene in this group the association remained significant (*p*-value 4.9 × 10^− 5^), indicating that the observed treatment-modifying signal is robust to adjustment for co-amplified genes within the region.

We additionally identified non-*EGFR* mutations that were significantly associated with progression and response on EGFR therapy, which may be interpreted as treatment modifiers. Such associations were discovered for 8 mutations (deletions in *SDHD*, *MYBL1*, *GATA3* associated with decreased progression; deletions in *RASA1* and *XRCC4* genes on arm 5q14 associated with increased response; amplification of *EP300*, *DMC1*, *XRCC6* on arm 22q13 associated with increased response, see Fig. 5). Further examination of these association revealed that for all but the *MYBL1* mutation, the association between somatic carrier and progression / response outcomes remains significant when we restrict the cohort to only *EGFR* carriers during time intervals where EGFR treatment was administered to the patients (*n* = 200, Additional file 2: Fig. S3, see Methods). We performed a conditional analysis for correlated mutations within these regions similarly to the described above. The interaction associations between treatment response and the top alteration in each region remained significant after adjusting for the other correlated genes (*SDHD* interaction p-value 8. x10^− 5^, *RASA1* interaction p-value 5.17 × 10^− 5^, EP300 interaction p-value 6.27e-05).

All the associations found indicate further improved outcomes for these mutation carriers while on EGFR treatment. These results suggest there is an epistatic effect between *EGFR* and these genes, enhancing the effectiveness of EGFR treatment. Importantly, we were able to replicate many of these results using an external dataset in the DepMap portal [39] and CTRPv2 [42–44] (See Sect.  5).

**Fig. 6:**
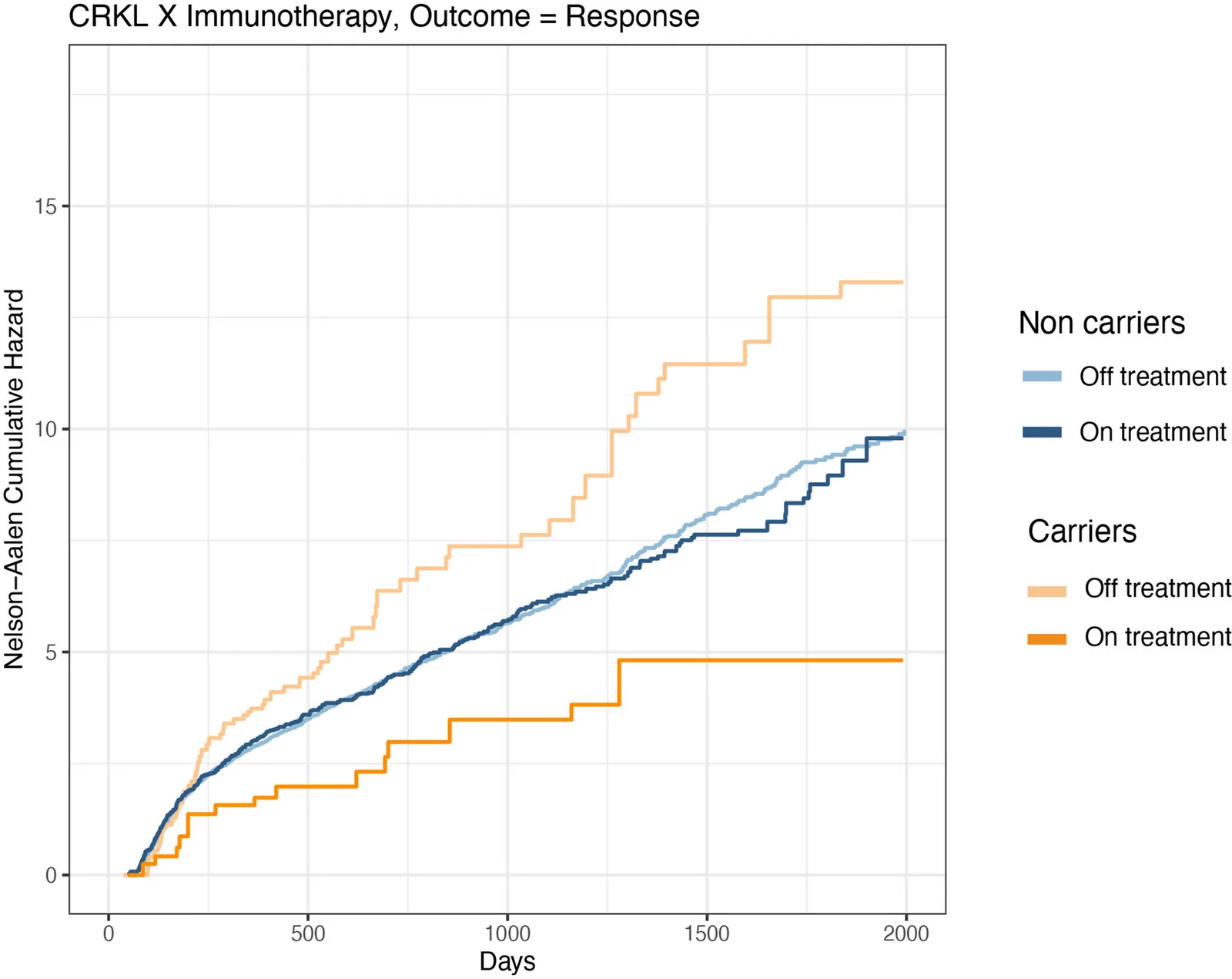
Cumulative hazard function of recurrent events for a sub-cohorts of carriers and non-carriers, on and off treatments. The plot represents the cumulative sum of recurrent response probabilities over time in our dataset. Patients are split into 4 groups – non-carriers (blue) and carriers (orange), while they are on treatment (darker colors) or off the treatment of interest (lighter colors). The X axis shows the days since diagnosis, and the Y axis shows the cumulative hazard function estimate as obtained by the Nelson Aalen estimate. While non-carriers of *CRKL* amplification have similar response rate on and off immunotherapy, carriers have lower response rates compared to non-carriers under the influence of immunotherapy drugs.

We highlight several interactions from the xAG-TD discovery set, which may be supported by or connected to previous findings in the literature. *LIG4* SNV carrier status interacted with chemotherapy to increase response rates (HR = 4.01 for response; p-value 1 × 10^− 4^, FDR p-value 1.5 × 10^− 2^). This may relate to a previous finding that *LIG4* mutation increases the sensitivity of platinum based chemotherapy including Cisplatin and Carboplatin, leading to increased OS and PFS [56]. Another significant association was detected between *CRKL* amplification associated with reduced rates of response to immunotherapy treatment (HR 0.45, p-value 1.36 × 10^− 4^, FDR p-value 0.02). This result corresponds to previous reports about the role of *CRKL* in mediation of anti-PD-1 resistance in liver cancer [57], and its expression being a predictive biomarker for anti-PD-1 therapy response in melanoma and breast cancer. We further examined the *CRKL*-immunotherapy interaction association by plotting the Nelson-Aalen cumulative hazard function plot (Fig. 6). Because the event type is response, in this plot, having a higher cumulative hazard is the more favorable outcome. For non-carriers, there seems to be a similar cumulative hazard when on and off immunotherapy. For example, at 1-year since diagnosis, the cumulative hazard for response in non-carriers of *CRKL* amplification is 2.88 (SE 0.12) and 3.02 (SE 0.22) off and on immunotherapy, respectively. On the other hand, *CRKL* amplification carriers have a differential response to immunotherapy, with a cumulative hazard estimation of 3.73 (SE 0.5) and 1.56 (SE 0.56) off and on immunotherapy. This result may suggest that *CRKL* carriers might be considered for alternative treatments to immunotherapy or PD-1 inhibition alone.

We performed a sensitivity analysis by repeating the analysis only on the cohort of advanced stage patients (stages 3–4). As this reduces sample size, we were not able to assess all associations, but out of 18 associations tested, all were nominally significant (p-value < 0.05) and in the same direction of effect in the advance stage cohort (Additional file 1: Table S4).

### Validation of xAG-TD associations using the DepMap Portal and CTRPv2

We sought to validate the associations identified by xAG-TD using data from the DepMap portal [39] and CTRPv2 [42–44] - external, open-access resources of cancer cell line dependencies. Among their datasets, DepMap includes results from PRISM assays 40], which measure the effects of drugs and compounds on cell line viability by quantifying the log fold change (LFC) in cell line abundance, while CTRPv2 provides independent drug sensitivity profiles based on dose–response curves summarized by area-under-the-curve (AUC) metrics.

**Fig. 7:**
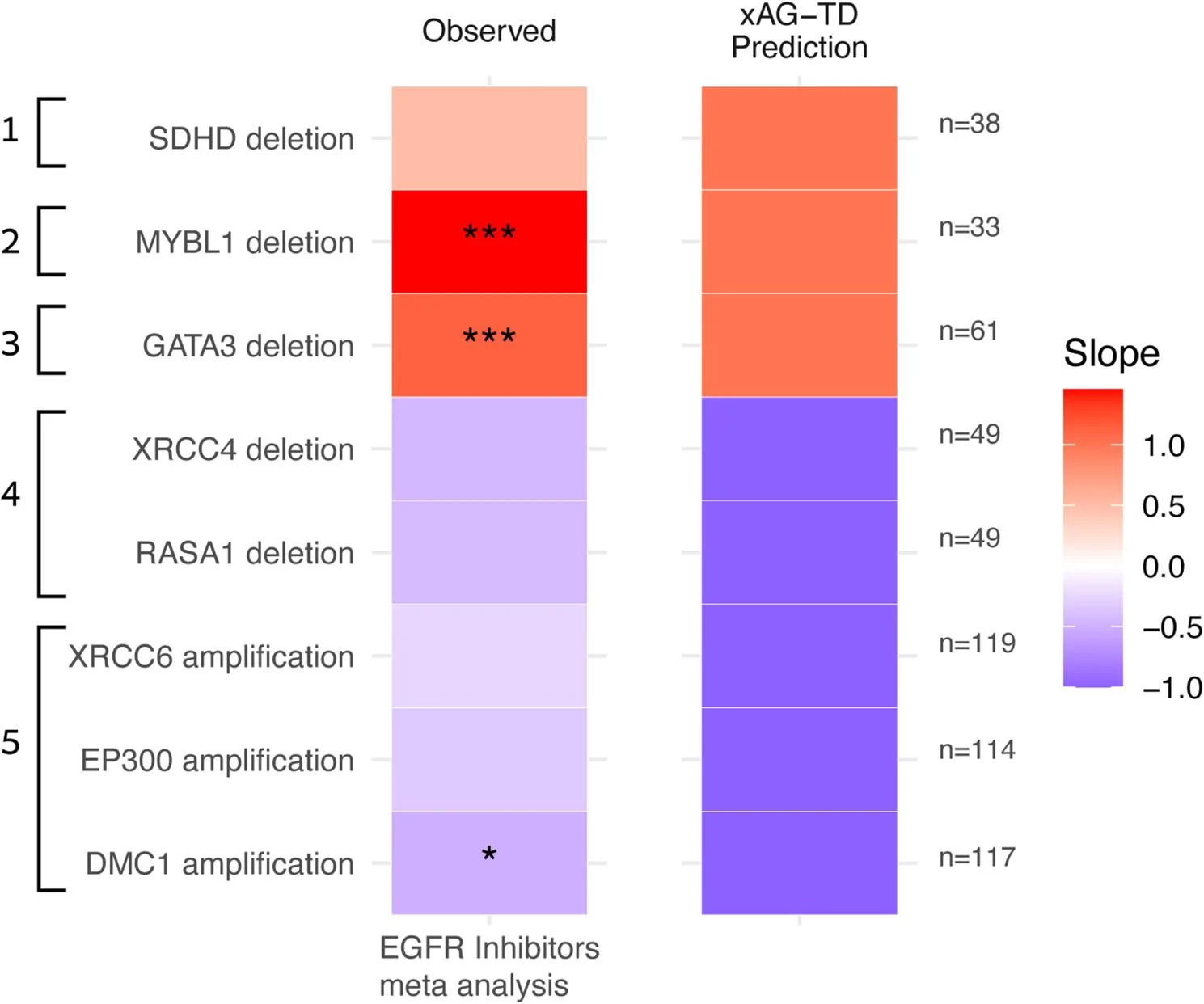
EGFR inhibitors meta-analysis validation of modifier genes using DepMap PRISM assay data. Each tile shows the slope and *p*-value from a fixed-effect meta-analysis assessing the association between gene-level copy number alterations (rows) and response to six EGFR inhibitors. The column on the right shows the predicted effect direction from the xAG-TD model as reported in Sec 4. Colors reflect the slope of a linear regression between continuous copy number and log fold change (LFC) in the PRISM assay (red = reduced sensitivity with higher copy number; blue = increased sensitivity). Asterisks indicate the statistical significance of the sided p-value: *** for Bonferroni significant, and * for p-value < 0.05

First, we focused on the response to six EGFR inhibitors in DepMap cell lines stratified by the copy number variations previously identified by xAG-TD as potential treatment modifiers. For each gene–drug pair, we calculated the one sided p-value and the direction of the slope from linear regression. A positive slope indicates that higher copy number is associated with higher log fold change (LFC) in cell line abundance after drug treatment, corresponding to reduced sensitivity to the drug. We further calculated a pan-drug slope and *p*-value using a fixed effect meta-analysis model (Fig. 7, Additional file 2: Fig. S4). Although eight associations were initially identified, some genes were found to be highly correlated and located in close genomic proximity. This reduced the analysis to five distinct gene sets: deletion in *SDHD*, deletion in *MYBL1*, and a deletion in *GATA3*; amplifications of *XRCC6*, *EP300*, and *DMC1*; and deletions in *XRCC4* and *RASA1*. 2 out of 5 of the gene groups (*GATA3* and *MYBL1*) showed a Bonferroni significant effect in the pan-drug meta analysis, and in a third group there was a nominally significant effect for one of the genes (DMC1). Notably, the signal for *GATA3* replicated in 50% of the individual drug tests, and each of the gene groups was nominally significant in at least one of the individual drug tests or in the meta-analysis (See Additional file 2: Fig. S4).

We next performed a replication analysis for these associations using the CTRPv2 dataset (See Additional file 2: Fig. S5). For the amplification in *XRCC6*, *EP300* and *DMC1* we found all to have a pan-drug meta analysis significant effect for the AUC-copy number correlation, matching the direction predicted by xAG-TD and observed in DepMap. The *GATA3* deletion was significant independently for some of the drugs (one sided p-values Gefitinib 3.7 × 10^− 3^ and Afatinib 4.36 × 10^− 3^) as well as for the pan-drug meta analysis. We were unable to test the *SDHD*, *MYBL1* mutations, and the group deletion in *XRCC4* and *RASA1* due to not having enough carriers in the data.

We additionally attempted to replicate the remaining associations identified by xAG-TD in Sects.  3 and 4 using these datasets. Only one mutation–drug pair was testable in both DepMap and CTRPv2 (the *TP53* SNV interaction with gemcitabine), and this association was not significant in either dataset. The *CDH1* deletion interaction with pemetrexed was testable in DepMap, and the *RAC1* amplification interaction with ALK inhibitors was testable in CTRPv2; however, neither showed significant evidence of replication. The remaining associations could not be evaluated due to insufficient numbers of mutation carriers among screened cell lines or lack of availability of the corresponding compounds in these resources. In particular, interactions involving immunotherapy agents are not testable in in-vitro cell line screens by design, due to the absence of an immune microenvironment.

In total, for both DepMap and CTRPv2 replications, the observed replication rate is significantly greater than expected by chance (binomial p-values 6.25 × 10^− 4^ and < 1 × 10^− 3^ for CTRPv2 and DepMap respectively). Several factors may explain the lack of replication for some gene–drug associations. The xAG-TD analysis was based on patients treated with EGFR inhibitors, most of whom harbored *EGFR* mutations, whereas DepMap and CTRPv2 cell lines are largely *EGFR* wild-type. Additionally, xAG-TD models all EGFR inhibitors as a single group, but individual drugs may differ in their effects. As a result, not all gene associations will replicate across every drug tested.

### Time dependent outcomes allow for novel discoveries of somatic mutation affecting the disease course

Finally, we sought to compare AG-TD model results with those obtained from classic Cox analysis using overall survival (OS) and progression-free survival (PFS) as outcomes. For this analysis we use PFS event time as the earlier of either survival or time of the first longitudinal progression event. For each mutation available we ran two Cox models: OS and PFS – and two AG-TD analysis for longitudinal outcomes, without treatment interactions: progression and response. For the response model, we restrict to time periods of active treatment. The number of carriers and not-carriers included in the analysis varies by gene and ranged from 20 to 462 carriers and 510–1618 non-carriers.

We first sought to evaluate the correlation between the logHR obtained for the same mutation by the four different models (Additional file 2: Fig. S6). A strong correlation between the logHR values of two models indicates that the signal and effect sizes detected for the same biomarker are consistent across those models. Across 1082 somatic mutations tested, there was a significant correlation (0.62, p-value 1.15 × 10^− 115^) between the logHR obtained in the OS and progression models. Response displayed a negative correlation with OS (-0.15, p-value 3.4 × 10^− 6^) and a weaker negative correlation with progression (-0.08, p-value 1.5 × 10^− 2^). Simulations using the observed estimation error showed that the modest observed response-OS correlation is consistent with a true, deattenuated underlying correlation of less than − 0.25 (see Methods and Additional file 1: Table S5). From these results, we see that progression and OS are related but not identical outcomes whereas response is distinct from both.

**Fig. 8:**
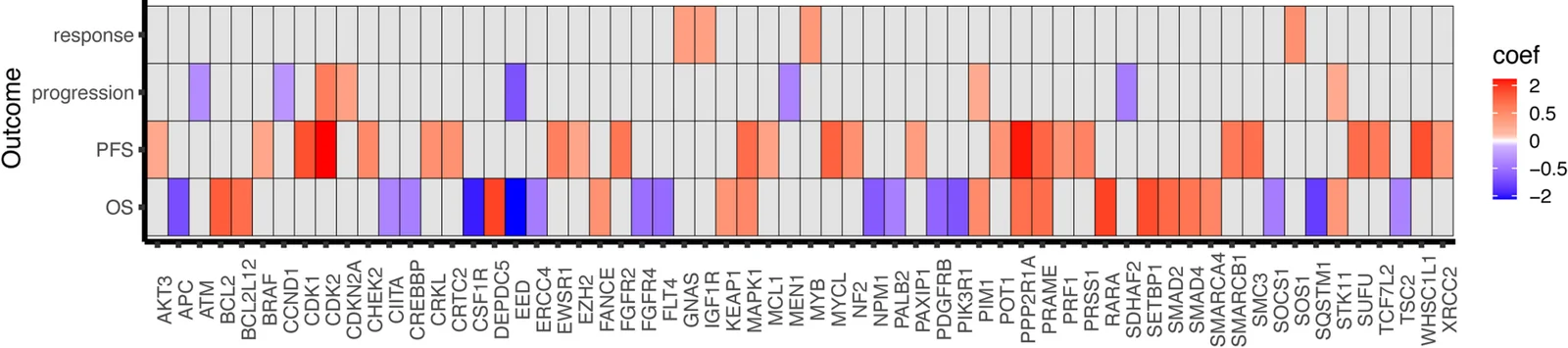
Significant associations detected between somatic mutations and OS, PFS, progression or response. The X axis shows the gene in which the mutation was found, and the colors show the logHR of the somatic mutation effect. All associations displayed are significant at FDR < 0.1 level

Next, we examine the mutations that showed significant associations (FDR < 0.1) in any of the models (Fig. 8). Overall, the OS outcome identified more significant associations than other models, however there are 9 associations that are only detectable using the AG-TD analysis for progression and response. Three associations were discovered for both progression and OS: *EED* gene amplification was associated with lower risk for OS and progression events (HR 0.34, SE 0.36 for progression ; HR 0.5, SE 0.19 for OS), *STK11* SNV was associated with increased risk for both progression and OS (HR 1.5, SE 0.09 for progression ; HR 1.3, SE 0.06 for OS) and *PIM1* amplification was associated with increased risk for both outcomes (HR 1.64, SE 0.13 for progression ; HR 1.3, SE 0.08 for OS). Four mutations were found to be associated with increased rate of response: *GNAS* SNV (HR 1.42, SE 0.1), *IGF1R* SNV (HR 1.41, SE 0.11), *MYB* amplification (HR 1.49, SE 0.1) and *SOS1* SNV (HR 1.58, SE 0.13), none of which were significant for either OS of PFS. We were not able to find previous evidence in the literature for these associations. It is of note that the PFS outcome model yielded 26 FDR significant associations, however only 3 of them were also FDR significant for OS, and overall, 12 of them were nominally significant (*p*-value < 0.05) for OS. These results demonstrate low replication of signal between these outcomes, contrary to results of a previous study [28]. To formally evaluate whether the degree of overlap exceeded chance expectation, we performed a hypergeometric enrichment test across pairs of models. OS (30 hits) and progression (9 hits) shared 3 mutations (*p*-value = 0.0014), and OS (30) and PFS (26) shared 3 (*p*-value = 0.033), both more than expected at random. By contrast, PFS and progression shared only 1 mutation (p-value = 0.20), and overlaps involving response were null (*p*-value = 1.0), confirming that response is largely distinct from both OS and PFS. To evaluate the sensitivity of this analysis to patient stage heterogeneity, we repeat it in the sub-cohort of advanced stage patients (stages 3–4). Out of the 69 FDR significant mutations, 54 were testable in the advanced stage sub-cohort, out of which 53 were marginally significant (*p*-value < 0.05) and all had the same direction of effect (the Pearson correlation between logHR is 0.985). Finally, we note that our interaction model, xAG-TD, identified nearly as many associations as the OS model, highlighting the potential of incorporating treatment interactions for association discovery.

We used simulations to explore possible scenarios leading to associations being detected by one of the models but not others. We developed a simulation framework encompassing a range of genetics models (see Methods). Our analysis showed that when genetic influences on OS are fully mediated through tumor growth, the recurrent progression model is the most effective, followed by PFS (Additional file 2: Fig. S7). In contrast, under a scenario where somatic mutations directly impact OS, bypassing tumor progression, the OS model outperforms both PFS and recurrent progression models (Additional file 2: Fig. S7). Notably, our simulations reveal that different genes may be identified by different models under various conditions, aligning with the experimental findings presented in Fig. 8. Under another framework, we tested a scenario where the somatic mutation is uncorrelated with progression or death but is linked to delayed diagnosis, possibly due to specific exposures or prevalence in a specific community (see Methods). In this case, the AG-TD model properly controlled the confounder, and OS reported false positives (Additional file 2: Fig. S8, see Methods). This result can be related to previous findings that successful cancer drugs often do not target genes associated with low OS [23].

The X axis shows the gene in which the mutation was found, and the colors show the logHR of the somatic mutation effect. All associations in displayed are significant at FDR < 0.1 level.

## Discussion

In this study, we present a novel approach for time-dependent survival analysis aimed at identifying associations between somatic mutations and patient outcomes. We focus on longitudinal signal related to tumor growth or shrinkage, rather than relying solely on overall survival (OS) and progression-free survival (PFS), for several reasons. First, longitudinal growth and shrinkage aligns more closely with what clinicians are optimizing and monitoring in their daily practice, providing a detailed, dynamic and relevant metric for research. Second, it allows to leverage the rich, underutilized data found in electronic health records (EHR), which can provide valuable insights that traditional metrics might miss. Third, it enables the identification of mutations that exert their effects at specific times during the disease course, for example, by interacting with specific treatments, offering a more nuanced understanding of how genetic changes influence patient outcomes over time. Lastly, using longitudinal signal may overcome the limitations associated with aggregating effects from various factors on patient OS and PFS outcomes. We applied our approach, AG-TD, to time-dependent data from the Dana-Farber Cancer Institute (DFCI) electronic health records (EHR).

We successfully detected somatic mutations that influence disease progression and treatment response and could not be detected with traditional OS and PFS analysis. Furthermore, by incorporating time-dependent indicators for treatments, we detected significant somatic mutation-by-treatment associations with progression or response outcomes. Each of these interactions represents a testable hypothesis regarding treatment-specific genetic effects that warrant further experimental and clinical evaluation. These hypotheses could be further evaluated and explored as part of the ongoing effort to improve and develop precision oncology. While our study confirmed expected somatic mutation-by-treatment interactions associations, such as the significant interaction between EGFR treatment and *EGFR* mutation, it also uncovered novel associations not previously reported in the literature.

Our study has several limitations that should be explored in future research. Firstly, our analysis was limited to NSCLC data from a relatively small sample at DFCI. Future studies should explore associations in other cancer types and larger cohorts to enhance new discoveries or replicate findings in other cancer types. Importantly, NSCLC comprises multiple histologic subtypes, but sample size limitations prevented further stratification of the analyses. Additionally, our model treated each mutation independently, overlooking potential co-occurrences and gene interactions, as well as tagging non-causal effects from long CNVs spanning multiple genes. While we validated some of the results in a multi-gene model, a joint fit model should be explored, once a bigger sample is obtained. Furthermore, while we included relevant covariates in our analysis, the possibility of unmodeled covariates influencing the observed associations cannot be ruled out. In particular, clinically relevant factors such as ECOG scores, comorbidity burden, and smoking were not available for all patients and thus were not modeled, and their omission may contribute to residual confounding in the estimated associations. Another important potential source of bias is within-patient correlation of recurrent events. Although we used patient-level cluster-robust standard errors to account for this dependence, coefficient estimates may still be biased if strong residual correlation among event intervals remains after covariate adjustment. Therefore, caution should be exercised in interpreting the results, and further investigation is needed to establish causality.

An important limitation of this study is that outcomes were derived from NLP models, and any directional misclassification could in principle introduce bias. However, given the very high discrimination of the NLP models used (AUROC 0.95–0.98), such errors are expected to be infrequent and largely non-differential, making substantial bias unlikely and suggesting that any residual impact would be conservative rather than inflating associations. The NLP models used to extract progression and response events were validated within the DFCI data environment, and any applications to other health systems would require independent validation. Similarly, treatment exposure was inferred using a carry-forward window of up to 12 months to address incomplete medication documentation, leading to potential misclassification of true off-treatment windows as exposed. However, a sensitivity analysis using a shorter window (up to 100 days) yielded highly concordant results, indicating the findings are robust to this modeling parameter.

One unexpected result was the lower number of associations detected in the AG-TD progression and response models compared to the OS model. Initially, we anticipated that longitudinal outcomes would yield more associations; however, our findings suggest the opposite. Through simulations, we show that, under certain conditions, the OS model may produce false positives that the progression and response models avoid. Additionally, simulations reveal that the choice of genetic generative models can influence the discovery power of each model. In addition, it is probable the sparsity of our longitudinal data, as well as its binary and noisy nature reduces our power compared to the OS analysis. Finally, while the DFCI OncoPanel is not designed to capture prognostic genes affecting overall survival, it is designed to include known oncogenes, tumor suppressor genes and genes involved in cancer relates signaling pathways [29]. Previous research demonstrated some oncogenes and tumor suppressor genes may serve as prognostic biomarkers, thus it is possible the OncoPanel is enriched for OS prognostic genes. It is possible that running a similar experiment with exome sequencing outside the OncoPanel targets will yield a higher number of associations with progression and response outcomes. Overall, we conclude that while the models yield correlated results, they do not fully overlap, and provide a complementary set of findings that enhance the discovery potential. The modest yet statistically significant negative correlation between response and OS may suggest partial decoupling of the drivers of short term tumor-regression and long term mortality. A possible explanation is that response is a treatment-proximal phenotype that primarily reflects mechanisms governing acute tumor regression under the current therapy, whereas OS integrates additional processes including resistance evolution, prior/subsequent treatment exposures, and cumulative disease burden. Consequently, genetic alterations that enhance short-term regression, such as context-specific sensitivities or synthetic lethal interactions, may not translate proportionally into long-term survival benefit. This does not imply that response is irrelevant to survival, as time-dependent response remains significantly associated with longer survival in our data, but rather that the genetic determinants of regression and mortality overlap incompletely.

## Conclusions

We present AG-TD and xAG-TD as statistical frameworks for time-dependent survival analysis in cancer research, leveraging longitudinal tumor outcomes derived from EHR data. We demonstrate the ability of these frameworks to identify somatic mutations associated with disease progression and treatment response as well as somatic mutation-by-treatment interactions that are undetectable using conventional survival methods.

By harnessing rich longitudinal EHR data, these frameworks provide a promising complementary approach to traditional survival analyses for biomarker discovery in cancer research. Importantly, these methods offer insights into the complex interplay between somatic mutations, treatments, and patient outcomes, and provide a foundation for future research aimed at advancing precision oncology and improving patient care.

## Supplementary Information


Additional file 1.



Additional file 2.



Additional file 3.


## Data Availability

The individual level tumor sequencing and matching electronic health records cannot be made publicly available because the research participant consent does not include authorization to share identifiable data.The CTRPv2 and DepMap dataset used for this study are publicly available online: the CTRPv2 dataset is available at https://portals.broadinstitute.org/ctrp.v2.1/ and the DepMap dataset is availbale at https://depmap.org/portal/.

## Abbreviations

AG-TD: Andersen-Gill based Tumor Dynamics
AUC: Area under the curve
AUROC: Area under the receiver operating characteristic curve
CNB: Copy number burden
CNV: Copy number variation
DFCI: Dana-Farber Cancer Institute
EHR: Electronic health record
FDR: False discovery rate
HR: Hazard ratio
IQR: Interquartile range
LFC: Log fold change
MAF: Minor allele frequency
MCF: Mean cumulative function
NLP: Natural language processing
NSCLC: Non-small cell lung cancer
OS: Overall survival
PFS: Progression-free survival
SNV: Single nucleotide variant
TMB: Tumor mutational burden
