# Supplementary material for: Leveraging tumor dynamics to discover mutations influencing progression and treatment response for precision oncology
Source: Genome Med. 2026 Jun 17;18:129. doi: 10.1186/s13073-026-01695-x (PMC13523221; doi:10.1186/s13073-026-01695-x)
Supplement: Supplementary file 1 [file 13073_2026_1695_MOESM1_ESM.docx]

**Supplementary Tables**

**Table S1**

| **Category** | **Drugs** |
| --- | --- |
| EGFR targeted drugs | Erlotinib, Gefitinib, Afatinib, Osimertinib, Cetuximab, Panitumumab, Lapatinib, Tarceva, Iressa, Gilotrif, Tagrisso, Erbitux, Vectibix, Tykerb, Dacomitinib, Vizimpro, Poziotinib, Patritumab, Mobocertinib, Amivantamab |
| ALK targeted drugs | Crizotinib, Alectinib, Brigatinib, Ceritinib, Lorlatinib |
| Chemotherapy | Carboplatin + Pemetrexed, Cisplatin + Pemetrexed, Carbo + Paclitaxel, Pemetrexed, Paclitaxel, Docetaxel, Gemcitabine, Vinorelbine |
| Targeted drugs | Bevacizumab, Trametinib, Sotorasib, Dabrafenib, Adagrasib, Ramucirumab, Selpercatinib, Pralsetinib, Capmatinib, Tepotinib, Entrectinib, Larotrectinib, Repotrectinib, Tucatinib, Neratinib, Encorafenib, Binimetinib, Vemurafenib, Cobimetinib |
| Immunotherapy | Pembrolizumab, Nivolumab, Durvalumab, Atezolizumab, Ipilimumab |

**Table S2**

| **Gene** | **Treatment** | **logHR** | **HR** | **Pvalue** |
| --- | --- | --- | --- | --- |
| ATM | vinorelbine | 0.444318 | 1.559426 | 1.27E-02 |
| CARD11 | pemetrexed | 1.002357 | 2.724697 | 2.74E-04 |
| CDH1 | pemetrexed | 0.90317 | 2.467413 | 6.38E-05 |
| GNAS | pemetrexed | 0.679479 | 1.97285 | 5.30E-02 |
| TP53 | gemcitabine | 0.636636 | 1.890112 | 2.62E-02 |
| AKT3 | immunotherapy | 0.472405 | 1.603847 | 6.08E-04 |
| CDC73 | immunotherapy | 0.45449 | 1.57537 | 1.08E-03 |
| CRKL | immunotherapy | -0.851076 | 0.426955 | 3.02E-04 |
| DMC1 | EGFR treatments | 0.698758 | 2.011253 | 5.84E-04 |
| EGFR | EGFR treatments | -0.741843 | 0.476236 | 1.23E-09 |
| EGLN1 | immunotherapy | 0.652519 | 1.920373 | 2.39E-04 |
| EP300 | chemo + immunotherapy | 0.906076 | 2.474593 | 8.31E-07 |
| EP300 | EGFR treatments | 0.829197 | 2.291477 | 1.13E-05 |
| FH | immunotherapy | 0.433428 | 1.542537 | 1.97E-03 |
| GATA3 | EGFR treatments | -0.588268 | 0.555288 | 6.41E-04 |
| MDM4 | immunotherapy | 0.528634 | 1.696613 | 8.30E-05 |
| MYBL1 | EGFR treatments | -0.474504 | 0.622194 | 1.14E-03 |
| PIK3C2B | immunotherapy | 0.543971 | 1.722835 | 4.35E-05 |
| PTPN14 | immunotherapy | 0.632079 | 1.881517 | 4.09E-04 |
| RAC1 | ALK treatments | 1.33122 | 3.78566 | 1.04E-05 |
| RASA1 | EGFR treatments | 0.818259 | 2.266549 | 1.79E-06 |
| UBE2T | immunotherapy | 0.603329 | 1.828194 | 4.83E-04 |
| XRCC4 | EGFR treatments | 0.78259 | 2.18713 | 9.27E-06 |
| XRCC6 | EGFR treatments | 0.852265 | 2.344953 | 3.66E-04 |

**Table S3**

| **Gene** | **Mutation type** | **logHR** | **HR** | **Tx** | **P-value** | **FDR p-value** | **N carriers** | **N non carriers** | **outcome** |
| --- | --- | --- | --- | --- | --- | --- | --- | --- | --- |
| GATA4 | deletion | -1.033 | 0.355 | carboplatin + pemetrexed | 0.003751 | 0.031827 | 86 | 301 | response |
| NEIL2 | deletion | -1.761 | 0.171 | carboplatin + pemetrexed | 0.000502 | 0.010803 | 51 | 130 | response |
| NKX3.1 | deletion | -1.867 | 0.154 | carboplatin + pemetrexed | 0.000263 | 0.010803 | 54 | 127 | response |
| PTK2B | deletion | -1.711 | 0.180 | carboplatin + pemetrexed | 0.001297 | 0.018593 | 58 | 123 | response |
| SETBP1 | deletion | -0.919 | 0.3985 | carboplatin + pemetrexed | 0.005181 | 0.031827 | 60 | 327 | response |
| SMAD2 | deletion | -0.923 | 0.397 | carboplatin + pemetrexed | 0.004914 | 0.031827 | 61 | 326 | response |
| SMAD4 | deletion | -0.968 | 0.379 | carboplatin + pemetrexed | 0.003428 | 0.031827 | 62 | 325 | response |

**Table S4**

| Mutation type | Gene | Tx | HR | logHR | P-value | Outcome |
| --- | --- | --- | --- | --- | --- | --- |
| mut | GNAS | Pemetrexed | 2.846 | 0.334 | 2.48E-04 | progression |
| mut | TP53 | Gemcitabine | 1.776 | 0.325 | 2.11E-02 | progression |
| mut | ATM | Vinorelbine | 1.556 | 0.236 | 1.49E-02 | progression |
| del | CDH1 | Pemetrexed | 2.613 | 0.302 | 2.61E-06 | progression |
| mut | EGFR | EGFR | 0.465 | 0.183 | 1.57E-09 | progression |
| del | GATA3 | EGFR | 0.521 | 0.174 | 7.62E-04 | progression |
| del | MYBL1 | EGFR | 0.620 | 0.182 | 5.54E-04 | progression |
| amp | RAC1 | ALK | 3.276 | 0.295 | 7.02E-04 | progression |
| amp | PIK3C2B | Immunotherapy | 1.580 | 0.114 | 7.34E-04 | progression |
| amp | MDM4 | Immunotherapy | 1.548 | 0.114 | 1.38E-03 | progression |
| amp | AKT3 | Immunotherapy | 1.512 | 0.116 | 3.03E-03 | progression |
| amp | CDC73 | Immunotherapy | 1.468 | 0.112 | 5.64E-03 | progression |
| amp | FH | Immunotherapy | 1.488 | 0.119 | 6.75E-03 | progression |
| amp | UBE2T | Immunotherapy | 1.499 | 0.141 | 1.47E-02 | progression |
| amp | EGLN1 | Immunotherapy | 1.500 | 0.145 | 1.87E-02 | progression |
| amp | PTPN14 | Immunotherapy | 1.479 | 0.147 | 2.45E-02 | progression |
| del | RASA1 | EGFR | 1.849 | 0.242 | 4.35E-05 | response |
| del | XRCC4 | EGFR | 1.778 | 0.242 | 2.91E-04 | response |
| amp | DMC1 | EGFR | 1.923 | 0.305 | 3.83E-04 | response |
| amp | EP300 | EGFR | 2.079 | 0.272 | 1.27E-05 | response |
| amp | CRKL | Immunotherapy | 0.448 | 0.303 | 1.50E-04 | response |
| amp | ZNRF3 | Immunotherapy | 0.208 | 0.617 | 3.63E-05 | response |

**Table S5**

| **Pearson correlation (simulated)** | **power** | **Mean correlation detected** | **Correlation SD** |
| --- | --- | --- | --- |
| -1.00 | 1.000 | -0.69 | 0.02 |
| -0.75 | 1.000 | -0.51 | 0.02 |
| -0.50 | 1.000 | -0.34 | 0.03 |
| -0.25 | 1.000 | -0.17 | 0.03 |
| -0.10 | 0.572 | -0.07 | 0.03 |
