# Supplementary material for: Leveraging tumor dynamics to discover mutations influencing progression and treatment response for precision oncology
Source: Genome Med. 2026 Jun 17;18:129. doi: 10.1186/s13073-026-01695-x (PMC13523221; doi:10.1186/s13073-026-01695-x)
Supplement: Supplementary file 2 [file 13073_2026_1695_MOESM2_ESM.docx]

**Supplementary figures**

**Fig. S1**

**Fig. S1. Comparison of logHR of the equipoise and cross-chemotherapy xAG-TD analyses.**

Each point represents one mutation that was found to be nominally significant (p-value < 0.05) in the cross-chemo xAG-TD model. The x and y positions mark the logHR from the cross-chemo and equipoise xAG-TD models, respectively. The pearson correlation of the logHR values is 0.948, and 27 of the mutations were also nominally significant in the equipoise analysis (marked in red).

**Fig. S2**

**Fig. S2. Cohort TMB distribution**

Distribution of tumor mutational burden in the study cohort. The dashed line denotes the cohort mean TMB value used to define the high-TMB covariate.

**Fig. S3**

**Fig. S3. logHR obtained from the AG-TD model run on a sub cohort of EGFR carriers, while on EGFR treatment**

The x-axis shows the logHR obtained for being a carrier of the specific mutation (y-axis). Each mutation was tested separately. The cohort only includes EGFR mutation carriers, and only time intervals when EGFR treatment was administered.

**Fig. S4**

**Fig. S4. Validation of EGFR treatment modifier genes using DepMap PRISM assay data.**

**Each tile represents the association between a gene-level copy number alteration (rows) and sensitivity to an EGFR inhibitor (columns), measured by log fold change (LFC) in the PRISM assay. Colors reflect the slope of a linear regression between continuous copy number and LFC (red = reduced sensitivity with higher copy number; blue = increased sensitivity). Observed sided p-value < 0.05 are marked inside the tiles. The final column in the drug panel represents a fixed-effect meta-analysis across all drugs, and the separated column on the right shows the predicted effect direction from the xAG-TD model as reported in Section 4.**

**Fig. S5**

**Fig. S5. Validation of EGFR treatment modifier genes using CTRPv2 data.**

**Each tile represents the association between a gene-level copy number alteration (rows) and sensitivity to an EGFR inhibitor (columns), measured by the AUC value reported in CTRPv2 dose-response assays. Colors reflect the slope of a linear regression between continuous copy number and LFC (red = reduced sensitivity with higher copy number; blue = increased sensitivity). Observed sided p-value < 0.05 are marked inside the tiles. The final column in the drug panel represents a fixed-effect meta-analysis across all drugs, and the separated column on the right shows the predicted effect direction from the xAG-TD model as reported in Section 4.**

**Fig. S6**

**Fig. S6. logHR correlations between different outcome models**

Pairwise logHR correlation of the same genes under different outcomes. OS and progression outcome models are correlated, but not identical.

**Fig. S7**

**Fig. S7. Comparing discovery power of Progression, PFS and OS models under different genetic architectures.**

The top panels show the discovery power of the three outcome models under changing parameters. On the left panel, the non tumor-growth genetic effect is fixed at $\mu=0.2$, and the x-axis displays the changing values for the probability of a growth event in carriers and non-carriers. The right panel has $\Pr\left( growth | G=1 \right)=0.4$ and $\Pr\left( growth | G=0 \right)=0.3$, and the x-axis displays the different values of $\mu$ for carriers and non-carriers. The Y axis shows the discovery power, estimated by the proportion of genes deemed nominally significant in each model.

The bottom panel compares the overlap of discovery between the different model. The x-axis displays the 100 genes simulated with $\Pr\left( growth | G=1 \right)=0.4$, $\Pr\left( growth | G=0 \right)=0.3$ , $\mu|G=1=0.4$ and $\mu|G=0=0.2.$ For each model, we display the FDR corrected p-value in a blue color scale where darker colors are more significant. Non significant genes will not be colored.

**Fig. S8**

**Fig. S8. QQ-plots for p-values obtained by OS traditional model and AG-TD progression model.**

The two panels present the QQ-plots for the p-values obtained from the OS classic model (left) and the AG-TD progression model (right). In this simulation we use 5K patients and 100 repetitions. The somatic mutation is uncorrelated with either progression or death, but is linked to delayed diagnosis. The OS model shows significant inflation and falsely detects this signal, as delayed diagnosis implies shorter survival (when measured starting at diagnosis). The AG-TD model follows the expected p-value distribution and does not report any false positives as the rate of progression is not associated with the mutation.
