# Supplementary material for: Leveraging tumor dynamics to discover mutations influencing progression and treatment response for precision oncology
Source: Genome Med. 2026 Jun 17;18:129. doi: 10.1186/s13073-026-01695-x (PMC13523221; doi:10.1186/s13073-026-01695-x)
Supplement: Supplementary file 3 [file 13073_2026_1695_MOESM3_ESM.docx]

**Supplementary Text**

**Replication of previously reported associations**

Previous studies by Liu et al.^28,58^ , detected significant effects of the interaction between first-line treatments and somatic mutations patients carry on overall patient survival. We performed a similar analysis in our dataset. Due to differences in cohorts, not all mutations were assessed in both datasets. When considering all associations that were significant (FDR < 0.05, n=59) in the Liu et al.^28^ paper, we find there is a significant correlation (0.374, p-value 0.0034) between the logHR obtained in both datasets.
